# A Needlelike Nano-hydroxyapatite-Based Hydrogel Accelerates Critical Bone Defect Regeneration via Osteo-/Angiogenesis and Osteoimmune Regulation

**DOI:** 10.34133/bmr.0344

**Published:** 2026-04-02

**Authors:** Dingli Xu, Leidong Lian, Zhe Luo, Yanxue Dong, Chaonan He, Minghui Chu, Liangjie Lu, Weihu Ma, Kaifeng Gan

**Affiliations:** ^1^ Department of Orthopaedics, The Affiliated LiHuiLi Hospital of Ningbo University, Ningbo 315000, Zhejiang, China.; ^2^ Health Science Center, Ningbo University, Ningbo 315100, Zhejiang, China.; ^3^ Department of Orthopedics, Deqing People’s Hospital, Huzhou, Zhejiang, China.; ^4^ Ningbo No. 6 Hospital, Ningbo 315100, Zhejiang, China.

## Abstract

The management of critical-sized bone defects has attracted heightened interest due to its challenging nature. To date, numerous engineered tissues incorporating nano-hydroxyapatite (nHap) have been proposed; however, nHap continues to encounter limitations, particularly regarding its inadequate immunomodulatory effects on bone. Therefore, needlelike nHap (NnHap)-based scaffolds were fabricated using a polylactic acid derivative and carboxymethyl chitosan. We hypothesize that NnHap@CP can not only promote bone immunomodulatory effects and angiogenesis in human umbilical vein endothelial cells through M2 subtype polarization but also directly promote osteogenesis in rat bone-marrow-derived mesenchymal stem cells (rBMSCs). Furthermore, mass spectrometry was employed to determine that osteoprotegerin/RANK/RANKL may represent a potential signaling pathway through which NnHap@CP enhances the osteogenesis of rBMSCs. In our study, NnHap@CP demonstrated a satisfactory effect on M2 subtype polarization in macrophages and enhanced osteogenesis in rBMSCs, as observed in an in vitro study. We employed NnHap@CP for the in vivo examination of a rat model with cranial critical-sized bone defects. We discovered that NnHap@CP significantly enhances new bone regeneration and neovascularization, potentially serving as an innovative treatment strategy for critical bone defects.

## Introduction

Critical-sized bone defects resulting from trauma or tumors can lead to delayed union or nonunion, presenting a marked challenge for orthopedic surgeons. The incidence of bone nonunion is estimated to be approximately 8% for femoral shaft nonunion, 4.6% for tibial shaft nonunion, and around 16% for open fractures [1]. Currently, autologous bone grafts, bone transport, and the induced membrane technique are prevalent strategies for managing bone defects, demonstrating efficacy in clinical practice; however, they are constrained by inadequate supply and donor-site-related complications [2]. While satisfactory clinical recovery was attained in certain patients, complications and failures were also documented. Bas et al. [3] reported that 40 patients with fractures underwent bone transport, resulting in 4 cases of nonunion at the fracture site, 1 case of fixation failure, and 5 instances of infection (1 case of cellulitis and 4 cases of osteomyelitis). Furthermore, patients with bone nonunion may experience bone deformity, internal fixation failure, and osteomyelitis. Additionally, the average cost of nonunion treatment represents a substantial economic burden on society. Ekegren et al. reported that 315 patients were readmitted to the hospital for fracture-related events, including bone nonunion, delayed union, or malunion, with an average hospital cost per patient of US$14,957 [4,5]. A strategy that enhances bone regeneration and prevents nonunion of bones holds significant clinical importance.

Bone tissue engineering is considered a viable approach for addressing critical bone defects. Potential solutions include fibrous and nonfibrous materials, synthetic substitutes, and cell-based products. However, hydrogel scaffolds not only mimic the bone extracellular matrix but also offer suitable mechanical support and a foundation for cellular proliferation and tissue regeneration [6]. Hydrogel scaffolds are effective bioengineering materials capable of loading diverse medical agents, including stem cells, decellularized scaffolds, and advanced pharmaceuticals, which can activate and recruit endogenous cells (such as bone mesenchymal stem cells, vascular endothelial cells, and macrophages) to the defect site. Subsequently, these endogenous cells release cytokines that promote osteogenesis, angiogenesis, and bone immunomodulatory effects [7]. Numerous researchers have indicated that hydrogels exhibit remarkable clinical efficacy in regeneration, encompassing spinal cord injuries, wounds, alveolar structures, kidney issues, long-bone defects, and cartilage repair through the encapsulation of stem cells, nanomaterials, growth factors, and bioactive molecules [8,9]. Recently, nano-hydroxyapatite (nHap), a promising implant material with structural and biochemical properties similar to those of the mineral composition of bone, has garnered considerable attention as a potential bioactive material [10]. Moreover, Chatzipetros et al. [11] reported nHap/chitosan scaffolds for the treatment of critical calvaria bone defects in Sprague–Dawley rats, and satisfactory bone regeneration was observed from histological and histomorphometric results. Moreover, loading bioactive factors and drugs onto nHap composites has emerged as a promising strategy for bone defect repair. Zhang et al. reported that a multifunctional nHap/MXene scaffold prevents tumor recurrence and promotes bone formation, demonstrating a robust capacity for osteoinduction in bone marrow mesenchymal stem cells. Similarly, a peptide MY-1-loaded nHap was synthesized that regulates the synthesis and secretion of the extracellular matrix and promotes cell migration and osteogenic differentiation in the early stage of bone regeneration [12].

nHap and related hydrogels demonstrated effective bone defect repair through cell proliferation, adhesion, and osteoinduction while also enhancing osteogenesis and angiogenesis when drugs, cytokines, or RNAs are incorporated into nHap [13,14]. However, these loading methods were limited by drug-related complications, rapid loss, and high costs [15]. Currently, many researchers are focusing on bone immunomodulation [16], which mitigates inflammatory responses and promotes osteoblast differentiation during bone tissue remodeling [17]. Macrophages play a crucial role in bone immunomodulation by exhibiting both M1 and M2 phenotypes. M1 macrophage, an inflammatory phenotype, can secrete pro-inflammatory cytokines to recruit other immune cells and promote the clearance of cellular debris in the early inflammatory phase; for the M2 phenotype, some cytokines are produced to modulate the inflammatory response and promote cell differentiation in bone regeneration. To regulate macrophage polarization, certain studies suggest that encapsulating specific anti-inflammatory drugs or cytokines within nHap can activate osteoimmunomodulation. Zhou et al. [18] reported that a composite hydrogel containing methacrylate-silk fibroin/nHap was fabricated and found that the composite hydrogel promotes bone regeneration by inducing M2 macrophage polarization during transcriptomic analysis. Similarly, Yang et al. [19] designed a titanium coat with nHap loaded with immunomodulatory cytokines (interleukin-4 [IL-4] and interferon-γ [IFN-γ]) and observed sequentially controlled release of loaded IL-4 and IFN-γ, which induce macrophage polarization.

While loading specific anti-inflammatory drugs or cytokines into nHap may be a viable approach to enhancing bone regeneration by activating macrophage M2 polarization, limitations such as drug-related complications, rapid degradation, and high costs persist. Sadowska et al. proposed that macrophages may be responsive to the microstructural and textural characteristics of nanophase materials, suggesting that modifying these structural features could effectively activate osteoimmunomodulation. They discovered that needlelike nHap (NnHap) can enhance the up-regulation of M2-related genes (*CD206* and *IL-10*) in RAW cells. Furthermore, conditioned medium (CM) from RAW cells and NnHap enhanced the osteoblastic differentiation of osteoblastic cells [20]. The osteoinductive effect of NnHap was further analyzed. Cetin et al. developed composite bioscaffolds incorporating NnHap and quince seed mucilage, demonstrating that this bioscaffold significantly up-regulates osteogenic gene expression in human adipose-derived mesenchymal stem cells, indicating a robust osteoinductive capacity in vitro [21]. However, there were few studies on NnHap-related composites for the treatment of bone defects, and the exact mechanisms by which NnHap enhances osteogenesis and osteoimmunomodulation remain to be elucidated. The material synthesis is mostly based on admixtures, which cannot be controlled for release [22].

Herein, we propose the design and fabrication of an efficient composite hydrogel for critical cranial bone defects. Carboxymethyl chitosan (CMCS) serves as the scaffold, mercaptopropionic acid (MA)-functionalized NnHap functions as the bioactive component, and a polylactic acid derivative (PLAD) acts as the cross-link between CMCS and NnHap, resulting in the successful fabrication of the composite hydrogel (NnHap@CMCS). This treatment is designed to initiate bone defect repair by activating macrophage polarization at the injured site and releasing related factors that promote osteogenesis and angiogenesis. The multiporous structure of CMCS and PLAD provides space for the proliferation of mesenchymal stem cells and osteoblasts. The osteogenic potential of the NnHap@CP hydrogel was assessed at the cellular level through alkaline phosphatase (ALP) and Alizarin Red S (ARS) staining. The impact of macrophage polarization activation was analyzed via immunofluorescence, and the underlying mechanisms were evaluated through protein sequencing. In an in vivo study, Sprague–Dawley rats with a critical bone defect in cranial bone were used as model animals for a bone repair experiment. Hematoxylin and eosin (H&E) and Masson staining revealed that the NnHap@CP hydrogel exhibited significantly improved regeneration in the defects. Furthermore, a markedly higher expression of osteogenesis-related proteins was detected in immunohistochemical sections. Furthermore, the messenger RNA (mRNA) and protein expression levels of osteogenesis-related markers were assessed using real-time quantitative polymerase chain reaction (qRT-PCR) and Western blot (WB) analysis.

## Materials and Methods

### Preparation of NnHap

Calcium nitrate tetrahydrate and diammonium hydrogen phosphate were utilized according to Fujishiro et al. [23]. The calcium source was prepared by dissolving 2.36 g of Ca(NO_3_)_2_·4H_2_O in 100 ml of deionized water. Then, 100 ml of 0.1 mol/l C_10_H_14_N_2_Na_2_O_8_ was added and mixed. Subsequently, 0.792 g of (NH_4_)_2_HPO_4_, serving as the phosphorus source, was dissolved in 100 ml of deionized water. Following this, 50 ml of 0.1 mol/l urea (CH_4_N_2_O) was added and stirred for 30 min. The mixed solution was subsequently placed in a hydrothermal reactor at 150 °C and pH 6 for 10 h and then centrifuged at 10,000 rpm for 30 min. Finally, the product was washed thrice with anhydrous ethanol and dried in a vacuum oven.

### Preparation of the NnHap@CP hydrogel

**Fig. 1.**
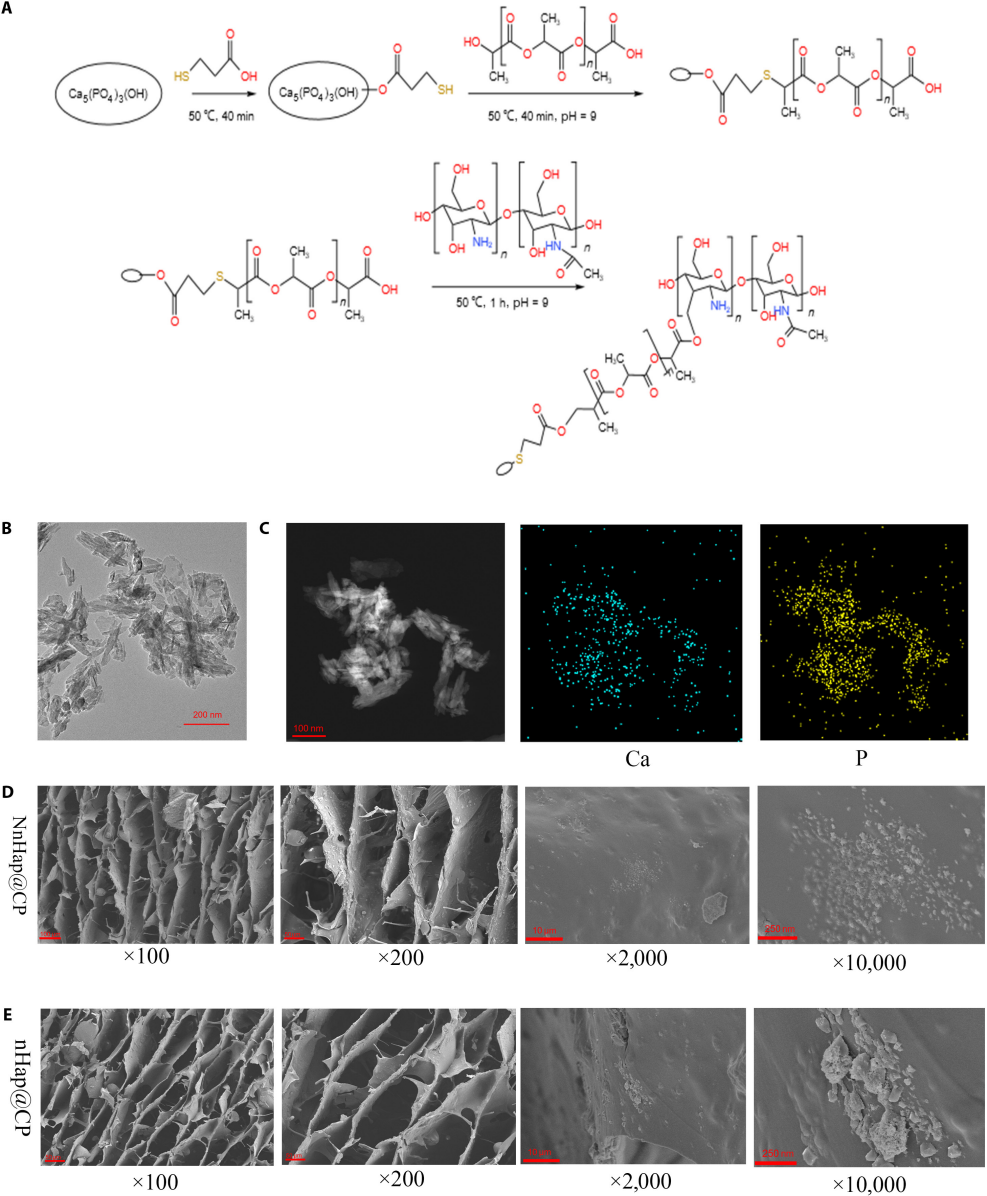
Characterization of the NnHap@CP hydrogel. (A) The schematic diagram of chemical equations for material synthesis; (B) the transmission electron microscopy (TEM) of needlelike nano-hydroxyapatite; (C) energy-dispersive spectroscopy (EDS) of needlelike nano-hydroxyapatite; (D) the scanning electron microscopy at ×100, ×200, ×2,000, and ×10,000 of the NnHap@CP hydrogel; (E) the scanning electron microscopy at ×100, ×200, ×2,000, and ×10,000 of the nHap@CP hydrogel. NnHap@CP hydrogel, needlelike nano-hydroxyapatite/carboxymethyl chitosan–polylactic acid derivative composite; nHap@CP hydrogel, nano-hydroxyapatite/carboxymethyl chitosan–polylactic acid derivative composite.

As shown in Fig. 1A, first, 0.2 g of NnHap was added to 20 ml of deionized water and stirred at 50 °C for 30 min. Adjusting the solution to pH 9.0 using an NH_4_OH solution, 0.4 g of MA (Sigma-Aldrich) was added and stirred for 3 h, resulting in the successful synthesis of MA-modified NnHap [24]. Second, 1.6 g of PLADs (Daigang Biology) was added to 10 ml of methylene dichloride (Aladdin), stirred for 10 min, and then mixed into the MA-modified NnHap solution. The mixture was stirred at 50 °C for 50 min. Later, 8 wt% CMCS (Sigma-Aldrich) was added to the solution and stirred at 50 °C for 30 min. Finally, 1 wt% NnHap@CP hydrogel (needlelike nano-hydroxyapatite/carboxymethyl chitosan–polylactic acid derivative composite) was successfully fabricated. To compare the materials’ characteristics, nHap@CP hydrogel (nano-hydroxyapatite/carboxymethyl chitosan–polylactic acid derivative composite) was also prepared using the same procedure.

### Characterization of the NnHap@CP hydrogel

The morphology and elemental composition of NnHap were determined using transmission electron microscopy (TEM) at 200 kV (JEOL F200, Japan). The sample for TEM was prepared by depositing 2 μl of the reaction solutions onto carbon-coated copper grids without dialysis or purification. Moreover, x-ray diffractometer (XRD) measurements were performed in an XRD (Bruker D8 Advance, Germany) [25]. The patterns were recorded in the region of 2*θ* 10° to 80°.

To evaluate the graft copolymerization percentage of MA and NnHap, ^1^H nuclear magnetic resonance (NMR) spectra were recorded on a 400-MHz NMR spectrometer (Bruker Advance Neo 400, Germany). The samples (15 ml) were dissolved in deuteroxide and placed in a 5-mm NMR glass tube (Wilmad-LabGlass Co., USA) for evaluation.

The surface morphology and elemental composition of NnHap@CP hydrogels were observed using scanning electron microscopy (GeminiSEM 360, Zeiss, Jena, Germany) and energy-dispersive spectroscopy [26]. NnHap@CP hydrogels were lyophilized for 24 h, and a layer of gold was sprayed on the surface to improve conductivity.

The chemical composition of the NnHap@CP hydrogel was analyzed using Fourier transform infrared spectroscopy (IRTracer-100, Shimadzu, Kyoto, Japan), with the absorbance mode set between 100 and 4,000 cm^−1^.

A compression test was performed using an electronic universal testing machine (China-UTM-5105). Cylindrical hydrogel samples (15 mm in diameter and 10 mm in height) were used for the compression test, with the compressive force applied at a rate of 0.5 mm/min until the sample fractured. Then, the load–displacement curves were transformed into stress–strain curves, and the maximum stress was taken from the turning point on the curve. The compressive modulus was calculated from the linear range in the stress–strain curve [27]. The elastic modulus (*G*′) and viscosity modulus (*G*″) were measured in a frequency range of 0.1 to 100 rad/s via a rheometer (Kinexus Lab+, NETZSCH, Germany). The stress amplitude and temperature were 1% and 25 °C, respectively.

Swelling and degradation rate were also measured [28]. Cylindrical hydrogels of identical dimensions were produced and subjected to lyophilization for 24 h (dry weight, *W*_0_), followed by the immersion of these samples in deionized water at ambient temperature. At each set time point, the surface water was removed before weighing at each measurement time (*W_t_*). The swelling rate was calculated via the following equation: swelling ratio = (*W_t_* − *W*_0_)/*W*_0_ × 100%. Each group underwent 3 repetitions for accuracy.

For the degradation test, identical cylindrical hydrogels were produced and lyophilized for 24 h (initial dry weight, *W*_0_). Subsequently, these samples were immersed in a collagenase II phosphate-buffered saline (PBS) solution at 37 °C. At each designated time point, the samples were rinsed with ultrapure water and weighed (*W_t_*) after drying. The degradation rate of NnHap@CP hydrogel was calculated using (*W*_0_ −*W_t_*)/*W*_0_ × 100%. Each group underwent 3 runs to ensure accuracy [29].

### Biocompatibility of the NnHap@CP hydrogel

The extraction of scaffolds was performed according to an International Organization for Standardization method (ISO 10993-12). Briefly, the scaffolds were incubated in a 100 mg/ml culture medium at 37 °C for 24 h. The supernatants were filtered through a sterilized 0.22-μm membrane filter (Biosharp, China) and stored at 4 °C for further use [30].

### Cell culture and seeding

Rat bone-marrow-derived mesenchymal stem cells (rBMSCs; Pricella, Wuhan, China) were cultured in alpha minimum essential medium (α-MEM) supplemented with 10% fetal bovine serum (FBS; Corning, USA) and 1% penicillin–streptomycin solution (Gibco, USA) in a humidified incubator with 5% CO_2_ at 37 °C. The culture medium was replaced every 2 d, and the adherent cells were cultured until they reached 80% to 90% confluence. Cells from passages 3 to 5 were harvested for subsequent experiments [31]. The RAW 264.7 cell line (Pricella, Wuhan, China) was used to explore the osteoimmunity of macrophages in vitro, which were cultured in Dulbecco’s modified Eagle medium (DMEM) with 10% FBS and 1% penicillin–streptomycin. The cells were cultured until they reached 90% confluence.

Human umbilical vein endothelial cells (hUVECs; Meisen, Zhejiang, China) were cultured in endothelial cell medium (ECM; ScienCell, Los Angeles, USA). The cells were cultured until 90% confluence.

### Cell cytotoxicity and live/dead staining tests

The cytotoxicity of NnHap@CP hydrogels was evaluated using a Cell Counting Kit-8 (CCK-8) assay. rBMSCs were seeded into a 24-well plate and incubated in either the scaffold extraction medium or standard medium within a 5% CO_2_ cell incubator at 37 °C for 3 and 5 d. Then, cell viability was assessed using the CCK-8 assay, and the optical density was measured at 450 nm using a microplate reader (Thermo Fisher Scientific, USA). Live/dead staining tests were also performed to assess the in vitro cytotoxicity of NnHap@CP hydrogels. rBMSCs (4 × 10^4^/well) were seeded into a 24-well plate and co-cultured with either the scaffold extraction medium or standard medium in a 5% CO_2_ cell incubator at 37 °C for 3 and 5 d. Later, rBMSCs were immersed in PBS containing 4 mM calcein-acetoxymethyl ester (calcein-AM) and 16 mM propidium iodide (PI) for 30 min (Elabscience, China). Moreover, the CCK-8 assays and live/dead staining tests of RAW 264.7 cells and hUVECs were also evaluated similarly at 3 and 5 d using the same procedure.

### The effect of NnHap@CP hydrogels on rBMSC osteogenesis

For ARS staining, 1 × 10^5^ rBMSCs were seeded into 6-well plates and divided into 4 groups: control, CP, nHap@CP, and NnHap@CP. The osteogenic differentiation medium (Oricell, China) was used to prepare the scaffold extraction medium, which was incubated with cells in all groups after they adhered to the surface of the 6-well plate. Before ARS staining, the cells were cultured for 21 d, and the medium was changed every 3 d. At 3, 5, 14, and 21 d, the entire medium was removed, and the cells were washed 3 times with PBS. They were then fixed with 4% paraformaldehyde at room temperature for 15 min and washed 3 times with PBS. After that, 1 ml of 0.2% ARS solution (Solarbio, China) was added to each well and incubated at room temperature for 15 min. The excess staining solution was removed, and the sample was washed 3 times with PBS. The precipitated cells were imaged and subsequently dissolved in 10% cetylpyridinium chloride for quantitative measurement at an absorbance of 562 nm [32].

For ALP staining and activity assay, rBMSCs (4 × 10^4^/well) were seeded into a 24-well plate and co-cultured with the extraction medium as aforementioned, after the cells had adhered to the surface of the 24-well plate. Before undergoing ALP staining and activity assay, the cells were cultured for 3, 5, 14, and 21 d, with the medium changed every 3 d. The culture media were aspirated and rinsed with PBS 3 times before the addition of 4% paraformaldehyde to fix the cells at room temperature for 20 min. Then, 300 μl of ALP staining working solution (Beyotime, China) was added to each well and incubated at room temperature for 30 min, protected from light. Finally, the cells were rinsed with deionized water to halt the color development and imaged.

The ALP activity assay was conducted after 3, 5, 14, and 21 d of incubation. The medium was removed, and the cells were washed 3 times with PBS. Then, 300 μl of radioimmunoprecipitation assay (RIPA) lysate was added. The suspension was centrifuged at 12,000 rpm and 4 °C for 5 min. The supernatant was obtained and incubated in a 96-well plate using the ALP activity assay working solution in a cell culture incubator for 30 min. Postincubation, the absorbance of the reaction mixture was measured at 405 nm using a microplate reader (Thermo Fisher Scientific, USA).

Osteogenesis-related mRNAs were evaluated by using a qRT-PCR assay (Table S1). Around 1 × 10^5^ rBMSCs were seeded into 6-well plates for 48 h and divided into 4 groups: the control, CP, nHap@CP, and NnHap@CP groups. Then, these cells were incubated with the scaffold extraction medium for 1 week. The total RNA from each sample was extracted according to the protocol provided with the RNA extraction kit (TransGen, Beijing, China). The extracted RNA was then reverse-transcribed into complementary DNA using the RT reagent kit (PrimeScript RT Master Mix, TransGen), and qRT-PCR assay was finally performed (Thermo Fisher Scientific, QuantStudio 5, USA). The mRNA levels of osteogenic genes, including recombinant bone morphogenetic protein 2 (*BMP2*), recombinant runt-related transcription factor 2 (*RUNX2*), and collagen type I (*COL-1*), were evaluated and normalized to the internal control, glyceraldehyde-3-phosphate dehydrogenase (*GAPDH*). The PCR primers are shown in Table S1.

Protein sequencing is regarded as a primary method for analyzing proteomics. To gain further insights into the proteomics of NnHap@CP and rBMSCs, liquid chromatography–mass spectrometry tandem analysis was employed to identify and analyze the protein constituents of rBMSCs. The identified proteins underwent systematic bioinformatics analysis, encompassing protein annotation, functional classification, and enrichment analysis. Immunofluorescence was used to confirm osteogenesis, while qRT-PCR and WB were used to confirm the underlying mechanism.

### The effect of the NnHap@CP hydrogel on macrophage polarization and inflammatory response

Immunofluorescence staining, flow cytometry, and qRT-PCR were used to evaluate the effect of the NnHap@CP hydrogel on macrophage polarization and inflammatory response. Pro-inflammatory macrophages (4 × 10^4^/well) were seeded into 24-well plates and incubated using various scaffold extraction media or the standard medium in a 5% CO_2_ cell incubator at 37 °C for 1 week. M1 and M2 macrophages were identified by immunofluorescence staining. For immunofluorescence staining, all samples were fixed with 4% paraformaldehyde for 15 min, after the medium of each cell was removed and the cells were washed 3 times. Then, 0.5% Triton X-100 (Solarbio, China) was used to permeate the cytomembrane, and bovine serum albumin (Solarbio, China) was used to protect proteins. Furthermore, the cells were incubated with inducible nitric oxide synthase (iNOS) antibody (Proteintech, China) and CD206 (Proteintech, China) at 4 °C overnight. Next, each well was washed thrice with 10× PBST (PBS with Tween 20; T1081, Solarbio, China). The cells were then processed with CoraLite 488-conjugated Goat Anti-Rabbit IgG (H+L) and CoraLite 594-conjugated Goat Anti-Rabbit IgG (H+L) for 1 h in the dark at room temperature. Finally, 4′,6-diamidino-2-phenylindole (DAPI; Beyotime, China) was used to stain nuclei, and the cells were photographed using an inverted fluorescence microscope (Leica DMi8, Germany). In order to quantify and analyze the proportion of M2-polarized cells, the flow cytometry were conducted. Precisely, pro-inflammatory macrophages (4 × 10^4^/well) were seeded into 24-well plates and incubated using various scaffold extraction media or the standard medium in a 5% CO_2_ cell incubator at 37 °C for 3 d. Then, cells were collected and centrifuged at 23,000 r/s and adjusted to 1 × 10^5^ cells/ml with PBS, fluorescein isothiocyanate-labeled antibodies against CD206 (Cat. No. 17-0869-42, Thermo Fisher Scientific, USA), and allophycocyanin-labeled antibodies against CD86 (Cat. No. MA562234, Thermo Fisher Scientific, USA) at 4 °C in dark. Flow cytometry was conducted by fluorescence-activated cell sorting (BD FACSVerse, USA), and the data were analyzed utilizing FlowJo (version X, BD, USA). What is more, flow cytometry was performed at 2 weeks in order to verify the stability of M2 polarization.

Similarly, qRT-PCR was used to analyze the expression of related mRNAs (pro-inflammatory genes: *IL-1β* and *IL-6*; anti-inflammatory genes: *IL-10* and *TGF-β*) in macrophages at 1 and 2 weeks.

### The effect of NnHap@CP hydrogel co-cultured macrophages on the osteogenesis of rBMSCs

The collection of CM was as follows: RAW 264.7 cells (1 × 10^5^ cells/well) were inoculated onto scaffolds in 6-well plates, with cells cultured in plates containing only DMEM serving as the control group. Then, the collected cultured media were filtered through a 0.22-μm filter to obtain CM after 3 d of incubation, and the CM was mixed with α-MEM culture medium in a 1:1 ratio. In this study, the groups were divided into the control, CP, and NnHap@CP groups.

Similarly, ARS staining, ALP staining, and ALP activity assay were performed at 14 and 21 d. ARS staining of rBMSCs was performed and cells were imaged at 14 and 21 d of culture with CM, categorized into the control, CP, and NnHap@CP groups. Subsequently, Alizarin Red S was dissolved using 10% cetylpyridinium chloride for quantitative measurement at 562 nm. Moreover, ALP staining and activity assay were also used to identify the osteogenesis of the rBMSCs cultured with CM after 14 d.

After the related signaling pathway was screened by mass spectrometry, the related mRNAs (osteogenesis related: *BMP2*, *RUNX2*, and *COL-1*; signaling pathway related: osteoprotegerin [*OPG*] and receptor activator of nuclear factor-κB ligand [*RANKL*]) were also evaluated by using qRT-PCR. Furthermore, these related proteins were also verified using WB. About 1 × 10^5^ rBMSCs were seeded into 6-well plates for 48 h and divided into 4 groups (control, CP, nHap@CP, and NnHap@CP). These cells were then incubated with CM for 1 week, with the CM being changed every 2 d. The RIPA lysis buffer solution (Solarbio, China) was used to soak the cells for 30 min, and the lysed samples were centrifuged at 12,000 rpm at 4 °C for 15 min in a 1.5-ml microcentrifuge tube. The supernatants were then collected. Then, the total protein concentration was detected using a bicinchoninic acid protein assay kit (Solarbio, China). The samples were loaded into sodium dodecyl sulfate–polyacrylamide gel electrophoresis gel for electrophoresis and transferred to polyvinylidene difluoride (Solarbio) membranes. The membranes were incubated with primary antibodies at 4 °C overnight before blocking in 5% milk for 1 h. These membranes were incubated with secondary antibodies (Proteintech, USA) after washing 3 times with Tris-buffered saline with Tween-20 (Solarbio). Finally, the blotting results were observed using an imaging system (Tanon 5200, Shanghai, China).

For short interfering RNA (siRNA) transfection, target-specific siRNA (si-OPG) and a negative control siRNA (si-NC) were transfected into rBMSCs cultured in 6-well plates. Cells were transfected with 20 μM siRNA using Lipofectamine 6000 transfection reagent (Beyotime Institute of Biotechnology) and then cultured for 48 h at 37 °C. The silencing efficiency was evaluated by qPCR. The si-NC used in the present study was a nontargeting control siRNA purchased from Shanghai GenePharma Co., Ltd., and its sequence was not disclosed by the manufacturer. Later, WB and PCR were performed to analyze the expression of RANKL, OPG, COL I, and RUNX2 after 1 week, which can testify whether inhibiting the RANKL/receptor activator of nuclear factor-κB (RANK)–OPG pathway will inhibit the osteogenic effect of NnHap@CP.

### The effect of NnHap@CP hydrogel co-cultured macrophages on the angiogenesis of hUVECs

To assess the effect of macrophage-conditioned CM on the angiogenesis of hUVECs, the scratch assay, the tube formation assay, and qRT-PCR were performed. For the scratch assay, hUVECs (1 × 10^5^ cells/well) were seeded into a 6-well plate and incubated with 5% CO_2_ at 37 °C for 48 h. Using a 1-ml pipetting spear head, a scratch was made along the central axis in every well [33]; after the scratches were successfully made, the cells were cultured with CM according to their group. The scratch site was photographed immediately using an inverted fluorescence microscope (Leica DMi8, Germany). The cells were then incubated with 5% CO_2_ at 37 °C for 12 and 24 h, and the photos were recorded at the same location.

The transwell assay was conducted to evaluate the effect of the NnHap@CP hydrogel on hUVECs’ migration. hUVECs were resuspended in ECM (ScienCell, Los Angeles, USA) devoid of FBS and positioned in the upper chamber, while ECM with various co-culture media, categorized into the control, CP, nHap@CP, and NnHap@CP groups, was placed in the lower chamber. After incubation with 5% CO_2_ at 37 °C for 48 h, the cells in the upper chamber were removed with a cotton swab. The remaining cells were fixed using formaldehyde and stained using crystal violet. Then, the cells were observed under an optical microscope, and the number of cells was quantified using ImageJ.

The tube formation assay was also conducted to evaluate the angiogenesis of hUVECs. A Matrigel matrix (Corning, USA) was thawed overnight in the refrigerator at 4 °C. The Matrigel matrix was then mixed with DMEM at a ratio of 1:5, as specified in the kit. The Matrigel matrix (200 μl/well) was laid on a 24-well plate on ice and incubated at 37 °C for 30 min. After washing the plate with DMEM, hUVECs (1 × 10^5^ cells/well) were seeded into a 24-well plate. Then, 300 μl of various CM were added into each well and cultured at 37 °C in 5% CO_2_ for 12 h. The microvascular formation was observed and imaged using an inverted fluorescence microscope (Leica DMi8, Germany). Furthermore, the expression levels of the angiogenic factors CD31 and vascular endothelial growth factor (VEGF) were assessed using qRT-PCR and immunofluorescence staining.

In order to further verify the effect of NnHap@CP in promoting hUVECs’ angiogenesis through M2 subtype polarization, we performed enzyme-linked immunosorbent assay (ELISA) to measure the concentration of VEGF and angiopoietin 1 in CM at days 7, 14, and 21. ImageJ (National Institutes of Health, USA) was used to analyze the rate of scratch healing, which was determined by the migration ability of cells, the number of vascular branches, and the point of tube formation in the assay.

### In vivo animal study

All animal feeding and operation procedures complied with the relevant laws and were authorized by the Ethics Committee of Ningbo University. Twenty-four 6-week-old male Sprague–Dawley rats were purchased from the Laboratory Animal Center of Ningbo University and were randomly divided into 4 groups (control, CP, nHap@CP, and NnHap@CP groups). Then, the rats were anesthetized by inhalation of isoflurane (Sigma-Aldrich, USA), and the fur on their scalps was shaved and sterilized. A 4-cm incision was made along the midline of the cranium, exposing the periosteum. Subsequently, a critical-sized cranial bone defect measuring 6 mm in diameter was created using a drill in each Sprague–Dawley rat [34]. The defects treated with CP, nHap@CP, and NnHap@CP were categorized into the CP, nHap@CP, and NnHap@CP hydrogel groups, respectively. The defects without any treatment were set as the control group. Then, the periosteum and skin were sutured respectively. Euthanasia by CO_2_ asphyxiation was performed on the rats after 4 and 8 weeks. The whole cranial bone was obtained, and the bone defects were investigated using a micro-computed tomography (micro-CT) system (Venus Micro CT VNC-102, Jiangsu, China). The new bone volume (BV), new bone volume/tissue volume (BV/TV) ratio, and trabecular bone number (Tb.N) were calculated after 3-dimensional (3D) reconstruction.

Histological and immunohistochemistry analyses were also conducted. The cranial bone samples were decalcified using 20% EDTA solution for 1 month. The samples were then fixed in 10% formaldehyde for 1 week. Next, the samples were dehydrated through a series of ethanol gradients, cleared with xylene, and embedded in paraffin. Sections approximately 4 mm thick were prepared and stained with H&E and Masson’s trichrome. Finally, each piece was observed and imaged using an inverted fluorescence microscope (Leica DMi8, Germany). Immunohistochemistry analysis was performed using OPG (1:100, R1609-4, HuaBio), CD31 (1:100, AF0077, Affinity), and COL-1 (1:100, AF7001, Affinity).

To evaluate whether stable M2 polarization persists in vivo. Immunofluorescence of skull specimens was performed according to the manufacturer’s instructions (Zhongshan, Beijing, China). Polyclonal antibody against S100A8 (1:100, Proteintech, USA) was applied. The sections were incubated with rhodamine (tetramethylrhodamine isothiocyanate)-conjugated goat anti-rabbit IgG (Sigma, USA) for 1 h at room temperature. Nuclei were stained with a DAPI solution (Sigma, USA) for 5 min. Images were captured using an optical microscope (Zeiss, Germany) (duplicate samples: 3; number of tests: 3).

Additionally, Matrigel plug angiogenesis was performed to analyze the angiogenesis of NnHap@CP scaffolds in vivo. Matrigel plugs were prepared by mixing with 50 U/ml heparin and the designated scaffolds according to group (control, CP, nHap@CP, and NnHap@CP groups). The mixture was subcutaneously injected into the back of the Sprague–Dawley rats. After 7 d, the rat were euthanized using CO_2_, and the Matrigel plugs were isolated. The plugs were photographed to document gross morphological changes. Moreover, the hemoglobin content was extracted from the plugs using 0.04% ammonium hydroxide solution. Then, the hemoglobin levels were quantified by measuring the absorbance at 540 nm using a spectrophotometer [35].

### Statistical analysis

Analysis and presentation of data were conducted using IBM SPSS Statistics version 26 (SPSS Inc., USA) and GraphPad Prism version 10 for Windows (GraphPad Software, USA). The normality of the data distribution was verified using the Shapiro–Wilk test. The differences between groups at 2 specific time points were compared by 2-way analysis of variance (ANOVA), and the differences between groups at a specific time point were compared by a one-way ANOVA. Statistical significance was denoted as *P* < 0.05.

## Results and Discussion

### Characterization of NnHap

**Fig. 2.**
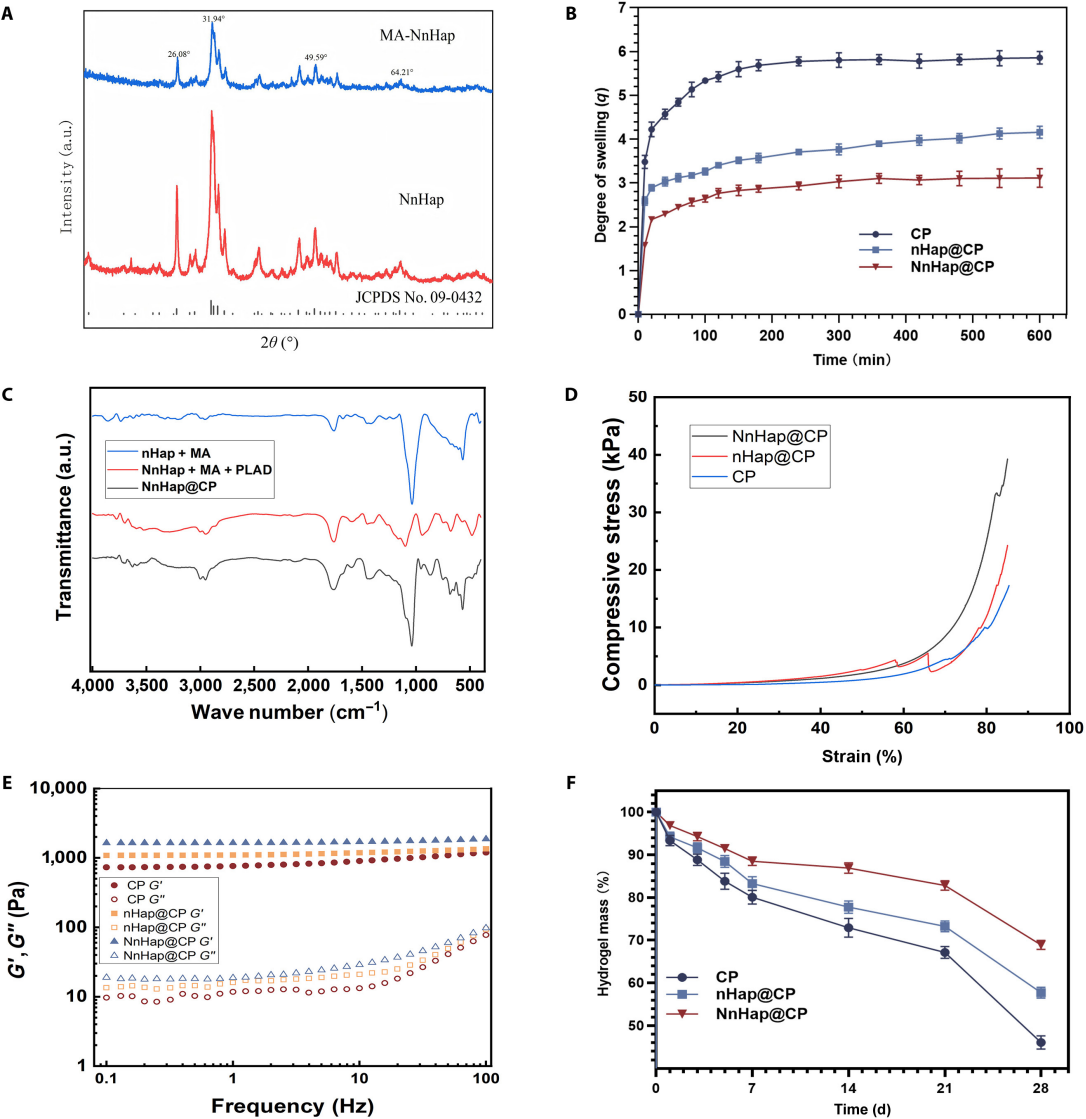
Characterization of the NnHap@CP hydrogel. (A) The x-ray diffractometer measurements of needlelike nano-hydroxyapatite and mercaptopropionic acid (MA)-modified needlelike nano-hydroxyapatite; (B) results for the swelling ratios of 3 scaffolds and (C) the Fourier transform infrared spectroscopy of all composites; (D) the compression test results of NnHap@CP, nHap@CP, and CP; (E) the rheological experiments of NnHap@CP and nHap@CP; (F) the results for the in vitro degradation ratios of 4 scaffolds. Results are represented as the mean ± SD of 3 independent experiments (*n* = 3). nHap, nano-hydroxyapatite; NnHap, needlelike nano-hydroxyapatite; CP, carboxymethyl chitosan–polylactic acid derivative; PLAD, polylactic acid derivative.

From Fig. 2A, the XRD spectra show that the obtained powders exhibit peaks similar to those of hydroxyapatite (JCPDS No.: 09-0432). The element mapping showed that Ca and P (representing hydroxyapatite) were present (Fig. 1C). TEM images (Fig. 1B) further confirmed that the synthesized powders had a needlelike shape.

### Microscopic morphological characteristics of the NnHap@CP hydrogel

Scanning electron microscopy micrographs revealed that both hydrogels possess a porous structure with an average pore size of approximately 150 μm (Fig. 1D and E). This porous structure can facilitate new bone in growth and neovascularization.

The element mapping of 2 hydrogels revealed that Ca and P (representing hydroxyapatite) were uniformly distributed throughout the scaffolds, indicating the successful incorporation of NnHap (Fig. S1).

Regarding the graft copolymerization rate, we conducted ^1^H NMR analysis of MA-modified NnHap (Fig. S2), and the grafting rate was found to be 43.8%.

### Fourier transform infrared spectroscopy

Figure 2C illustrates that the MA-modified NnHap exhibited pronounced absorption peaks at approximately 604 and 1,300 cm^−1^, indicative of the S–C covalent bond. In contrast, the MA-modified NnHap + PLAD exhibited significant absorption peaks at 3,000 and 1,750 cm^−1^, corresponding to a methyl group (–CH_3_) and a lipid bond (–COOH), respectively. Finally, the NnHap@CP exhibited pronounced absorption peaks near 3,300 cm^−1^, indicative of amidogen (–NH_2_), confirming the successful bonding of CMCS (Fig. 2C).

### Mechanical properties

Compression test results are demonstrated in Fig. 2D. The stress–strain curve and maximum compression modulus of scaffolds under a compressive process were analyzed. Compared to the nHap@CP hydrogel (24.26 ± 0.0841 kPa), the NnHap@CP hydrogel group achieved 36.45 ± 0.05926 kPa, demonstrating that the NnHap@CP hydrogel achieved better compressive properties.

Rheological experiments were performed to measure the *G*′ and *G*″ of scaffolds. The *G*′ values of NnHap@CP exceeded the *G*″ values at room temperature, indicating a solid-like hydrogel state (Fig. 2E). Moreover, the *G*′ values of NnHap@CP were higher than those of nHap@CP, implying that NnHap@CP achieved better mechanical properties.

### Swelling and in vitro degradation properties

Swelling and degradation properties are important characteristics of scaffolds for promoting tissue regeneration. Zhang et al. indicated that the swelling of a hydrogel typically results in volume expansion, which not only compromises the mechanical properties of the hydrogel but may also exert undesirable pressure on adjacent tissues when utilized in vivo. As shown in Fig. 2B, during the initial immersion (<150 min), the swelling ratio of all hydrogels increased dramatically. Over time (>300 min), the increase in the ratio of all hydrogels slowed down until they reached the final swollen state. CP scaffolds showed the highest swelling ratio during the entire immersion period compared to NnHap@CP and nHap@CP. The swelling ratios of nHap@CP were higher than those of NnHap@CP, indicating that NnHap demonstrated a superior antiswelling effect. The swelling behavior of a bone scaffold is critical for its clinical performance. An excessively high swelling ratio can generate detrimental stress on the surrounding bone tissue and impede new bone ingrowth, while a low ratio may not provide sufficient space for cell migration and nutrient diffusion [36]. Therefore, an ideal swelling ratio for bone regeneration is generally accepted to be in the range of 170.5% to 691.6% [37,38]. The swelling ratio of the NnHap@CP scaffold was measured to be 240% (Fig. 2B), which falls within this optimal range. This suitable swelling property, coupled with its interconnected porous structure, is believed to create a favorable microenvironment for osteoblast attachment and subsequent bone formation.

Wang et al. [39] reported that ideal bone repair materials should be fully biodegradable, with a degradation rate that matches the rate of bone tissue regeneration. In this study, the degradation test of the scaffolds was analyzed by immersing the samples in a collagenase II PBS solution at 37 °C. As the immersion time increased, all scaffolds showed loss of mass. After 28 d, the CP scaffold exhibited greater mass loss than the others, and the NnHap@CP scaffold showed a higher degradation ratio than the nHap@CP scaffold (Fig. 2F).

In our study, the NnHap@CP hydrogel exhibits superior degradation performance compared to nHap@CP hydrogels. The reason could be the following: (a) The needlelike structure of the needlelike nanomaterial may disrupt the uniformity of the hydrogel network, creating local stress concentration points, which promote crack propagation or phase separation and thereby accelerate degradation [40]. (b) The needlelike nanomaterial may increase the porosity of the hydrogel and expand the specific surface area, which result in accelerated degradation of the hydrogel [41]. Chen et al. [42] reported that scaffolds accelerate angiogenesis in the beginning, but with the acceleration of vessel network formation, the scaffold network hinders the process. Furthermore, the final biomechanical restoration in the NnHap@CP group confirms that the newly formed bone successfully assumed the load-bearing function. In contrast, nHap@CP group may have provided a prolonged but less bioactive template, resulting in a delayed and less robust bone healing response. Therefore, the degradation kinetics of NnHap@CP appear to be well calibrated to support an accelerated yet stable bone regeneration process.

### Cell cytotoxicity and live/dead staining tests

**Fig. 3.**
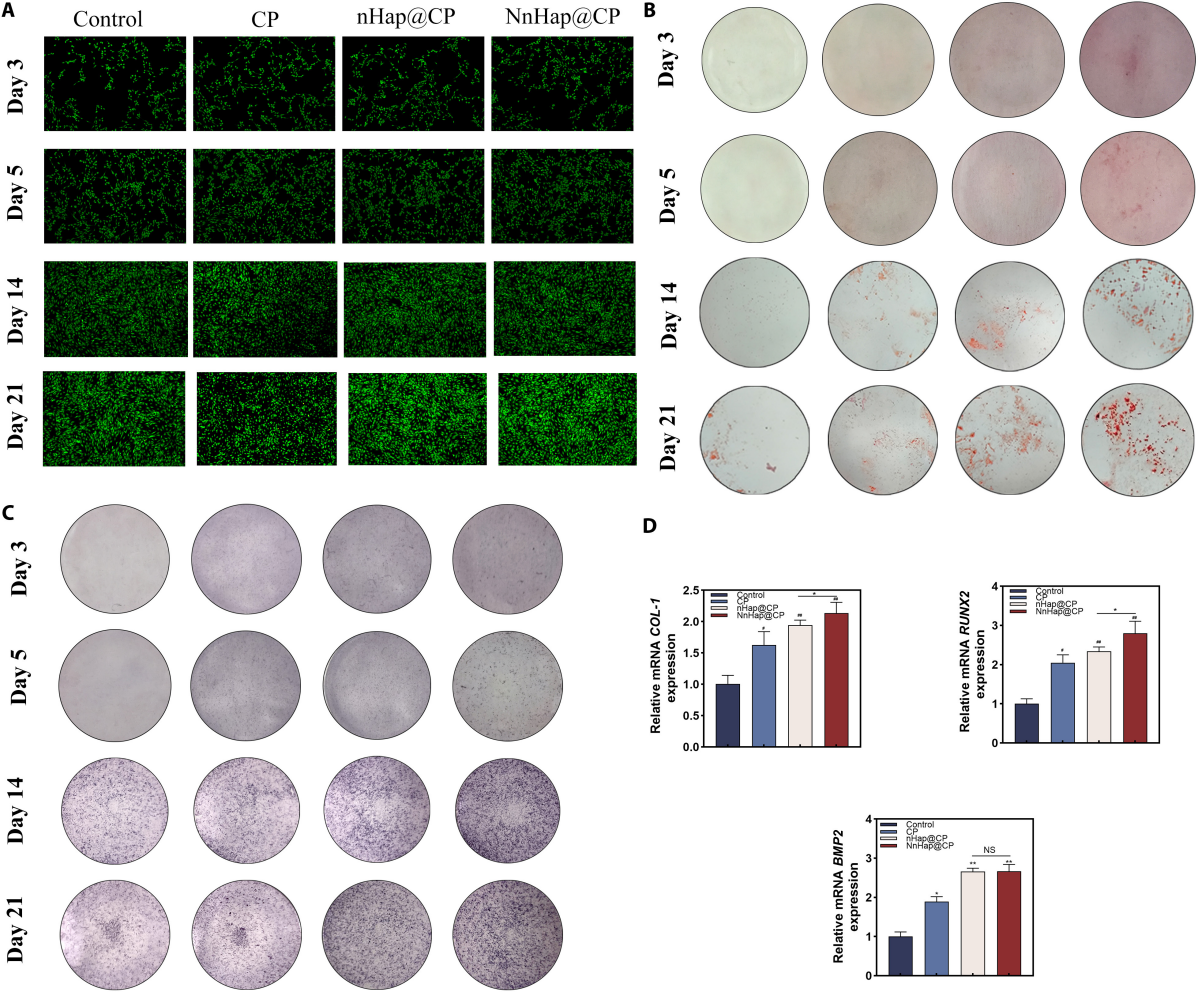
The effect of NnHap@CP hydrogels on rat bone-marrow-derived mesenchymal stem cell (rBMSC) osteogenesis. (A) The live/death staining assay of rBMSCs after 3, 5, 14, and 21 d; (B) the Alizarin Red S (ARS) staining of rBMSCs after 3, 5, 14, and 21 d; (C) the alkaline phosphatase (ALP) staining after 3, 5, 14, and 21 d; (D) the real-time quantitative polymerase chain reaction (qRT-PCR) of *BMP2*, *COL-1*, and *RUNX2* of rBMSC after 1 week. Results are represented as the mean ± SD of 4 independent experiments (*n* = 3); for statistical analysis, a one-way analysis of variance (ANOVA) test was used. Significant differences: *P < 0.05; ^#^*P* < 0.05; ^##^*P* < 0.01. NS, not significant.

In this study, calcein-AM/PI staining and CCK-8 were used. The results indicated that there was no significant decrease in cell viability for bone-marrow-derived mesenchymal stem cells (BMSCs) after 1 and 3 d of incubation (Fig. 3A). For CCK-8 analysis, there was no significant difference among the 3 groups after 1 and 3 d of incubation (Fig. S3A).

### The effect of NnHap@CP scaffolds on rBMSC osteogenesis

To evaluate the effect of NnHap@CP on rBMSC osteogenesis, we employed ARS and ALP staining to examine the osteogenic differentiation of rBMSCs after 14 and 21 d of co-culture with the scaffolds. The results of ARS and ALP staining showed that the NnHap@CP group achieved significantly better outcomes than the other 2 groups, the CP and nHap@CP groups, which achieved slightly better outcomes than the control group (Fig. 3B and C). Furthermore, the ALP activity assay and ARS quantitative analysis demonstrated analogous results (Fig. S3B and C).

Moreover, qRT-PCR technology was used to verify the osteogenesis-related mRNAs, such as *BMP2*, *RUNX2*, and *COL-1*. The results presented in Fig. 3D demonstrate that rBMSCs co-cultured with an extract of NnHap@CP effectively promoted their capacity for osteogenesis when compared to the nHap@CP and CP groups. Additionally, slightly better outcomes in mRNA expression were observed in the nHap@CP group compared to those in the control and CP groups. NnHap is efficacious in improving rBMSC osteogenesis capacity, a finding also reported by Sadowska et al. [20].

**Fig. 4.**
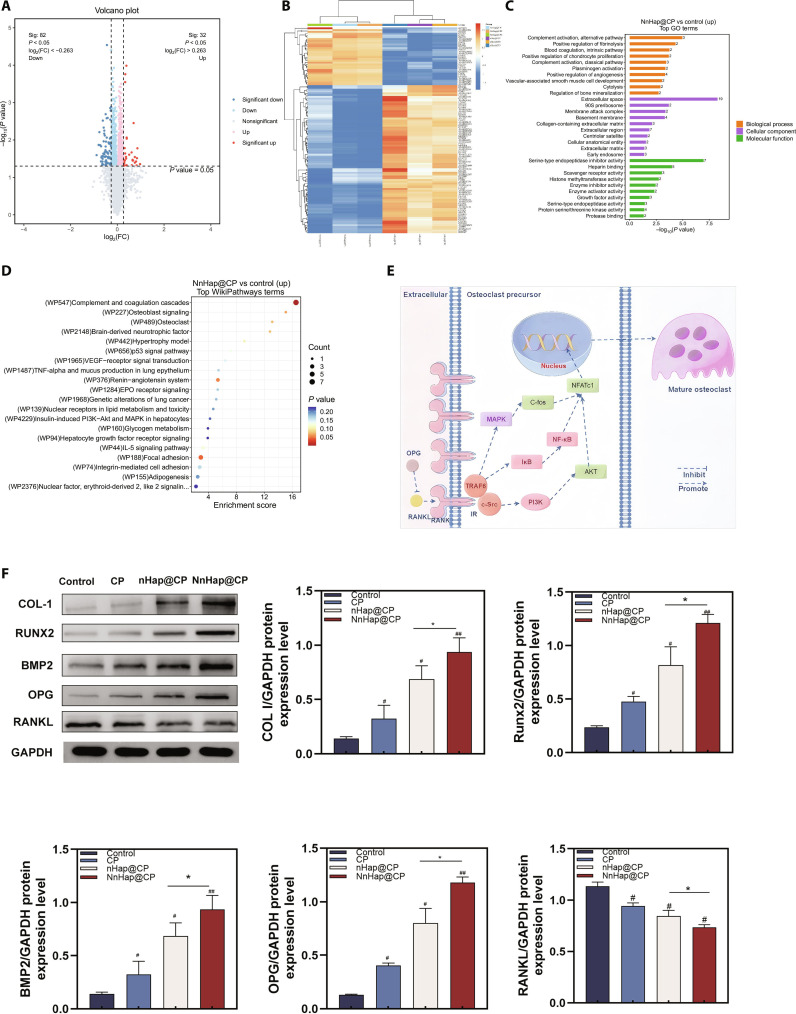
The results of protein sequencing and Western blot (WB) of rBMSCs. (A and B) The volcano and heat maps of the mass spectrometry of rBMSCs with conditioned medium (CM); (C) the Gene Ontology biological process category according to the volcano and heat maps; (D) the results of WikiPathways analysis; (E) schematic illustration of the mechanisms of NnHap@CP on rBMSCs; (F) WB of the osteoprotegerin (OPG)/receptor activator of nuclear factor-κB (RANK)/receptor activator of nuclear factor-κB ligand (RANKL) signaling pathway and quantitation analysis of the WB plot. Results are represented as the mean ± SD of 3 independent experiments (*n* = 3); for statistical analysis, a one-way ANOVA test was used. Significant differences: *P < 0.05; ^#^*P* < 0.05; ^##^*P* < 0.01. FC, fold change; Sig, significant; GO, Gene Ontology; GAPDH, glyceraldehyde-3-phosphate dehydrogenase.

To identify the potential mechanisms of the NnHap@CP scaffolds, protein sequencing was performed to determine the differentially expressed proteins in rBMSC cultures in the control and NnHap@CP groups for 5 d. Figure 4A and B show the volcano and heat maps, which were created to depict the differentially expressed proteins between the control and NnHap@CP groups. There were 32 up-regulated and 82 down-regulated proteins. In the Gene Ontology biological process category, biological processes, cellular components, and molecular functions were enriched (Fig. 4C). According to WikiPathways analysis (Fig. 4D), 4 signaling pathways were enriched, including osteoblast signaling (WP227), osteoclast (WP489), VEGF–receptor signal transduction (WP1965), and insulin-induced phosphoinositide 3-kinase–Akt and mitogen-activated protein kinase (WP4229) pathways. OPG and RANKL, as differentially expressed proteins, are significant regulatory molecules that have been demonstrated to influence bone regeneration in BMSCs [43]. The activation of the OPG/RANK/RANKL signaling pathway (Fig. 4E) can improve the function of osteoblasts [44]. To further indicate the protein expression, WB was also conducted to analyze the mechanism of the OPG/RANK/RANKL signaling pathway, which further indicated that the NnHap@CP group had the strongest effect on promoting the osteogenesis-related proteins (COL I, BMP2, Runx2, and OPG) expression, inhibiting RANKL protein expression (Fig. 4F).

In order to elucidate the role of the NnHap@CP scaffold’s active osteogenesis via the RANKL/RANK/OPG pathway, we used siRNA to inhibit OPG in rBMSCs. The PCR and WB of siRNA group reveal that transfection with OPG-targeted siRNA can effectively decrease the expression of OPG. NnHap@CP increased the expression of RUNX2, COL I, BMP2, and OPG, while transfection with OPG-targeted siRNA could decrease the expression of RUNX2, COL I, BMP2, and OPG (Fig. S4). These data confirmed that the NnHap@CP scaffold enhanced the osteogenesis of rBMSCs via the RANKL/RANK/OPG pathway in vitro.

As for OPG’s traditional role in osteoclasts, OPG is a soluble decoy receptor for RANKL, which binds and neutralizes RANKL and prevents RANKL from activating RANK on osteoclast precursors, thereby directly inhibiting osteoclast formation and activity and then suppressing bone resorption [45]. Moreover, the role of OPG in BMSC differentiation is that OPG produced by BMSCs acts on the cells themselves or their progeny to promote osteogenic differentiation while suppressing adipogenesis, via inhibiting the RANK/RANKL/OPG pathway [46]. Han et al. [47] reported that up-regulation of the OPG expression of bone marrow mesenchymal stromal cells can also increase Wnt1 and Runx2 expression via the paracrine effect of bone marrow mesenchymal stromal cells.

### The effect of NnHap@CP scaffolds on macrophages

Currently, macrophages are a type of initial cells that infiltrate bone defect regions, playing a pivotal role during bone regeneration. Some studies have demonstrated that M1 phenotype macrophages provoke the local pro-inflammation microenvironment, resulting in biomaterial implantation failure and bone repair impediment. In contrast, M2 subtype macrophages promote an anti-inflammatory microenvironment, which is conducive to osteogenesis [48]. As a consequence, the relation between scaffolds and macrophages was taken into account.

**Fig. 5.**
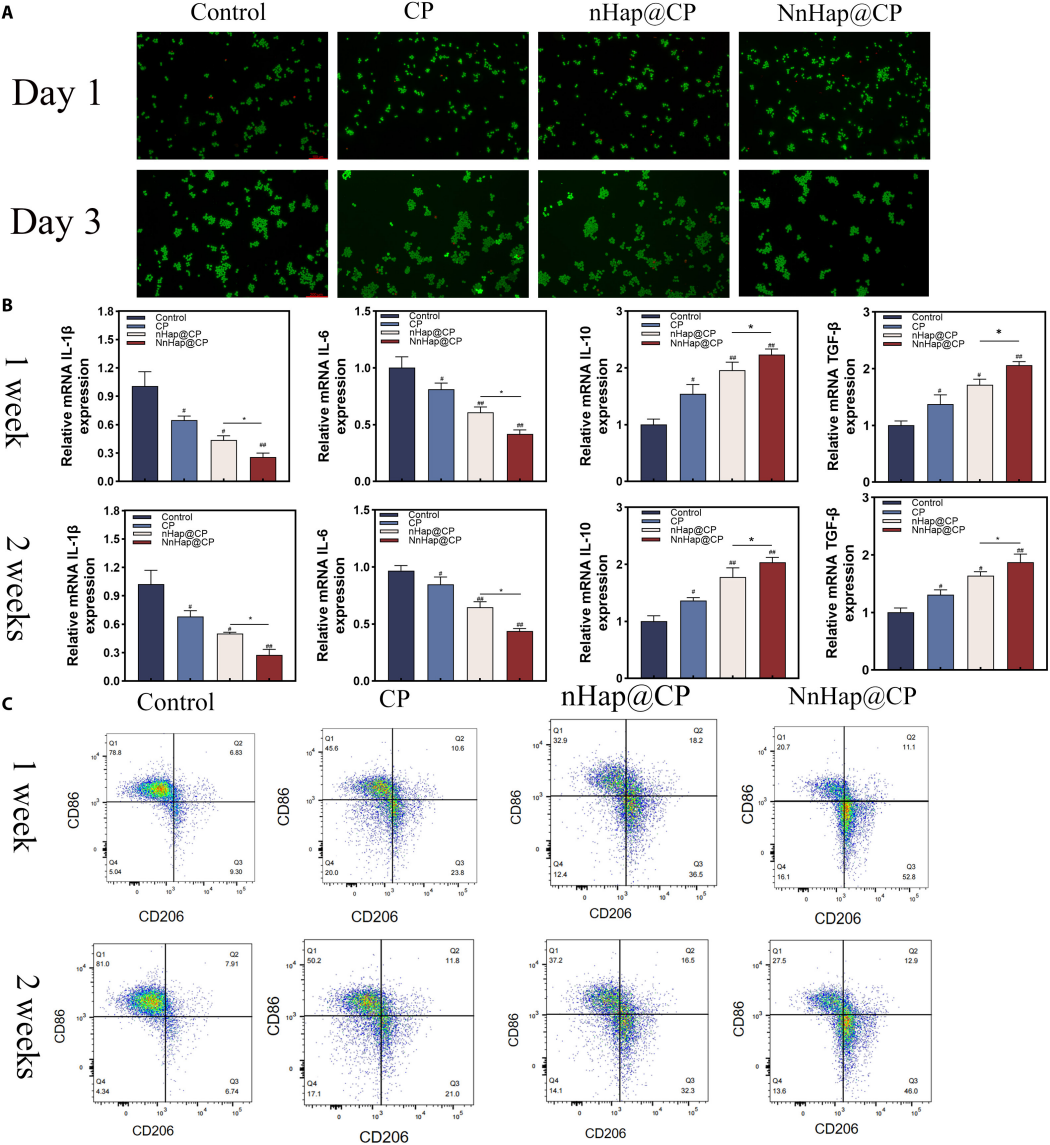
The effect of NnHap@CP scaffolds on macrophages. (A) The live/death staining assay of macrophages after 1 and 3 d (upper scale bar: 100 μm; lower scale bar: 200 μm); (B) the qRT-PCR of macrophages after 1 and 2 weeks; (C) results of the flow cytometry of macrophages at 1 and 2 weeks. Results are represented as the mean ± SD of 4 independent experiments (*n* = 3); for statistical analysis, a one-way ANOVA test was used. Significant differences: *P < 0.05; ^#^*P* < 0.05; ^##^*P* < 0.01. mRNA, messenger RNA; IL-1β, interleukin-1β; IL-6, interleukin-6; IL-10, interleukin-10; TGF-β, transforming growth factor-β.

First, the effects of all scaffolds on the cell viability of macrophages were evaluated by calcein-AM/PI staining and CCK-8 assay. The results indicated that there was no significant decrease in the cell viability of macrophages after 1 and 3 d of incubation (Fig. 5A). Similar outcomes were also observed in the CCK-8 assay (Fig. S5). The results of flow cytometry are shown in Fig. 5C. qRT-PCR was performed to investigate the effect of scaffolds on macrophages. As shown in Fig. 5B, the NnHap@CP group significantly reduced the expression of M1 phenotype macrophage markers (IL-1β and IL-6) compared to other groups. In addition, the nHap@CP group showed an improvement in the expression levels of M2 macrophage markers, including interleukin-10 (IL-10) and transforming growth factor-β (TGF-β). As shown in Fig. 5B and C, the qRT-PCR and flow cytometry of macrophages after 2 weeks showed that this M2 polarization was stable. The quantitative analysis of flow cytometry is shown in Fig. S6. Immunofluorescence was also performed to analyze macrophage polarization; we observed that the NnHap@CP group achieved the strongest fluorescence intensity of CD206 and the weakest fluorescence intensity of iNOS compared to other groups (Fig. S7A and B).

Although, as reported, nHap has shown effects on M2 phenotype polarization, NnHap achieved significantly better outcomes on M2 phenotype polarization than nHap. To identify the potential osteo-/angiogenesis effects of the NnHap@CP scaffolds, the effect of co-culture media on macrophages was further investigated in combination with rBMSCs and hUVECs [48,49].

### The effect of co-culture media of macrophages on rBMSC osteogenesis

The robust differentiation of BMSCs into osteogenic and angiogenic lineages in bone defect regions is influenced not only by the surface characteristics of implant biomaterials but also by the surrounding osteoimmune microenvironment, including the osteoimmunomodulation of macrophages [50]. We investigated the effects of macrophages and scaffold co-cultured media on the osteogenesis of rBMSCs.

**Fig. 6.**
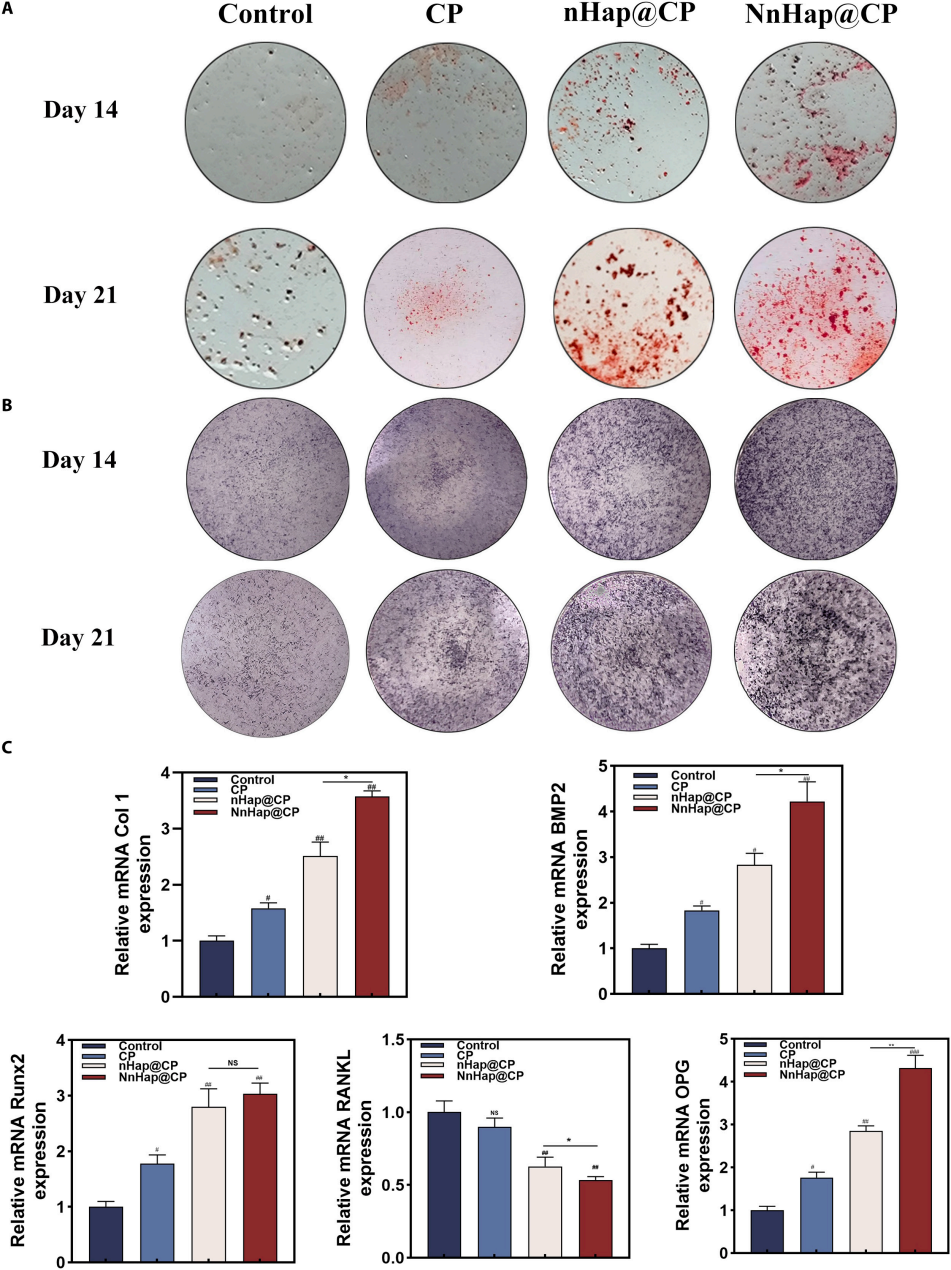
The effect of co-culture media of macrophages on rBMSC osteogenesis. (A) The ARS staining and (B) ALP staining of rBMSCs with CM after 14 and 21 d; (C) the qRT-PCR of rBMSCs after 1 week. Results are represented as the mean ± SD of 4 independent experiments (*n* = 3); for statistical analysis, a one-way ANOVA test was used. Significant differences: *P < 0.05; ^#^*P* < 0.05; ^##^*P* < 0.01.

ARS staining was used to analyze the rBMSCs’ mineralization capacity on days 14 and 21 (Fig. 6A), with the NnHap@CP group demonstrating the most mineralization. Moreover, quantitative analysis of ARS staining further indicated the mineralization capacity of NnHap@CP (Fig. S8A). Similarly, the results of ALP staining (Fig. 6B) and ALP activity assay (Fig. S8B) demonstrated that the group with the CM of NnHap@CP and macrophages had significantly higher ALP levels than the other groups.

Additionally, qRT-PCR and WB indicated that after 7 d of culture, the rBMSCs in the NnHap@CP group exhibited the strongest promotion of osteogenesis-related gene expression compared to the rBMSCs in the other 3 groups (Fig. 6C). Immunofluorescence staining and its quantitative analysis results (Fig. S9A and B) also showed that the COL I and OPG expressions in the NnHap@CP group were the highest among the groups, and the RANKL protein expression in the NnHap@CP group was the least among the groups.

Researchers believe that macrophages are crucial in bone defect regeneration by secreting various cytokines, including IL-10 and TGF-β, which enhance osteogenic differentiation, cell migration, and vascularization. The enhanced osteogenic differentiation capacity of rBMSCs incubated with CM may be attributed to NnHap@CP’s ability to elevate macrophage cytokine expression.

### The effect of NnHap@CP hydrogel co-cultured macrophage CM on the angiogenesis of hUVECs

**Fig. 7.**
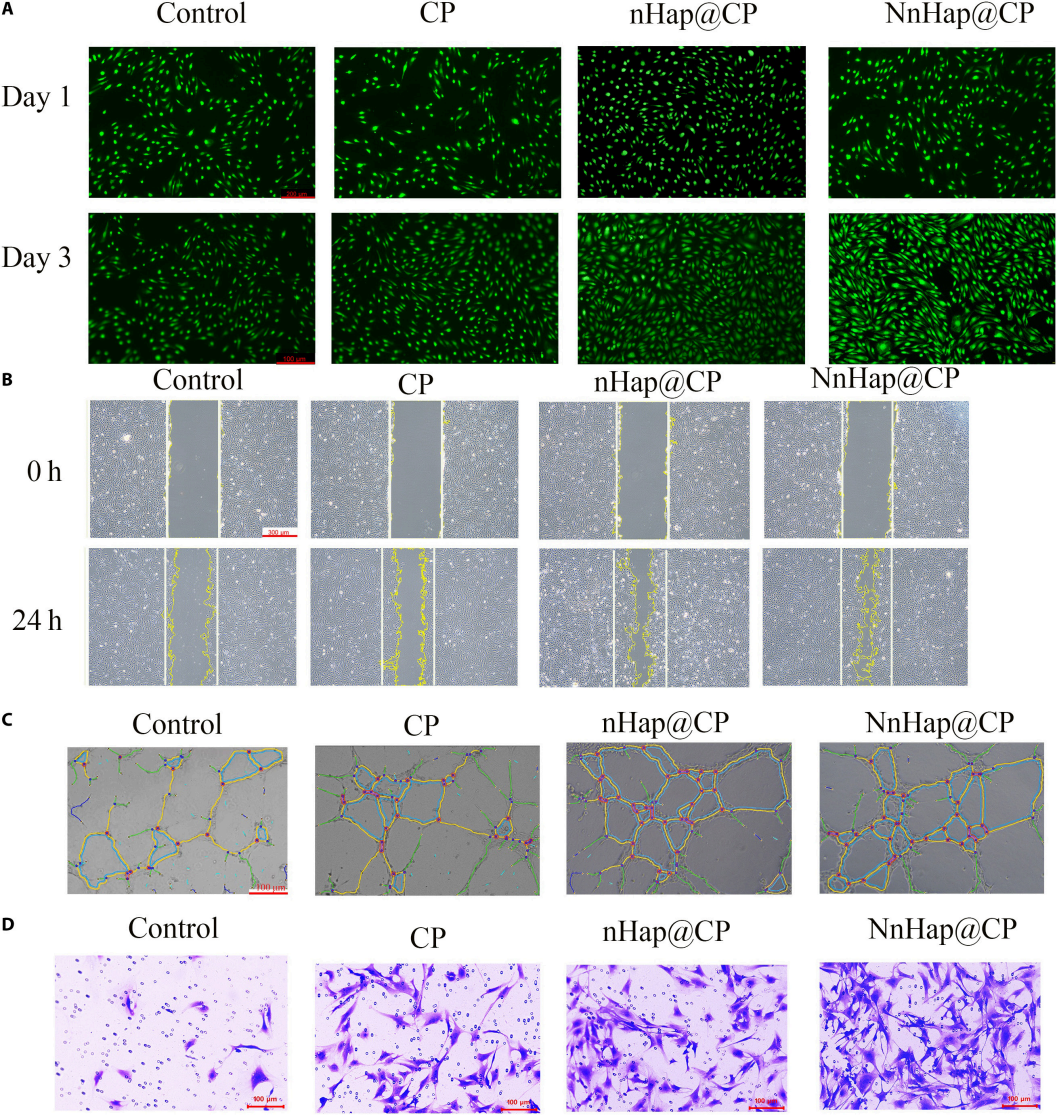
The effect of NnHap@CP hydrogel co-cultured macrophage CM on the angiogenesis of human umbilical vein endothelial cells (hUVECs). (A) The live/dead staining assay of hUVECs after 1 and 3 d; (B) the scratch assay of hUVECs at 0 and 24 h; (C) the tube formation of hUVECs; (D) the transwell assay of the 4 groups.

**Fig. 8.**
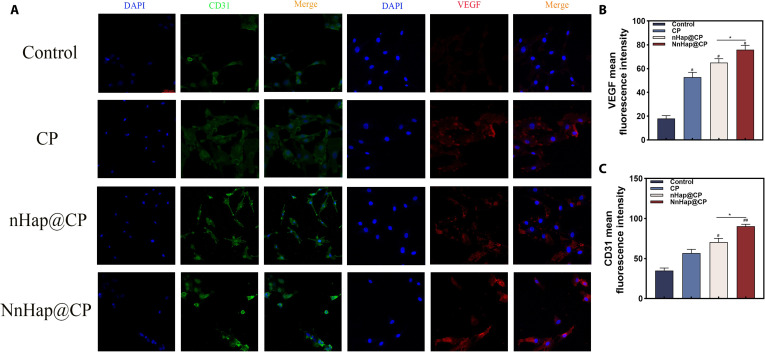
The effect of NnHap@CP hydrogel co-cultured macrophage CM on the angiogenesis of hUVECs. (A) The CD31 and vascular endothelial growth factor (VEGF) immunofluorescence staining of hUVECs at 1 week (scale bar: 100 μm); (B and C) the fluorescence intensity of CD31 and VEGF immunofluorescence staining. Significant differences: *P < 0.05; ^#^*P* < 0.05; ^##^*P* < 0.01. DAPI, 4′,6-diamidino-2-phenylindole.

Complete bone repair requires not only the osteogenesis of BMSCs but also neovascularization; therefore, the effects of hUVECs were examined. The cell viability of hUVECs was evaluated using calcein-AM/PI staining and the CCK-8 assay. The results indicated that there was no significant decrease in the cell viability of macrophages after 1 and 3 d of incubation (Fig. 7A). Similar outcomes were also observed in the CCK-8 assay (Fig. S10A). The migration ability of HUVECs co-cultured with various CM was studied (Fig. 7B). The NnHap@CP group achieved the greatest efficiency in enhancing the horizontal migration of hUVECs (Fig. S10B). Additionally, the tube formation assay was conducted, as illustrated in Fig. 7C, revealing that the NnHap@CP group exhibited significantly superior tube length and branch points compared to the other 3 groups (Fig. S10C and D). Moreover, a transwell assay was also conducted (Fig. 7D). Immunofluorescent staining of CD31 and VEGF on hUVECs was performed, as illustrated in Fig. 8A, and the quantitative results of immunofluorescent staining are shown in Fig. 8B and C. The results indicated that the NnHap@CP group exhibited significantly enhanced CD31 expression compared to the other groups.

The concentrations of growth factors of macrophage CM in the control, CP, nHap@CP, and NnHap@CP groups are shown in Fig. S10E and F. The NnHap@CP group achieved the highest concentrations of VEGF and angiopoietin-1 than the other 3 groups (*P* < 0.05). ELISA results reveal that the macrophage CM in the NnHap@CP group achieved significantly higher expression of VEGF and angiopoietin-1 at days 7, 14, and 21, which could provide direct evidence that M2-polarized macrophages are the source of the enhanced angiogenic effect in hUVECs.

The above findings suggest that NnHap@CP scaffolds not only enhance the migration capacity of hUVECs but also indirectly contribute to an increase in the angiogenesis ability of hUVECs via the mediation of a macrophage-regulated immunomodulation microenvironment (Fig. S11).

### In vivo study

The aforementioned in vitro studies demonstrated favorable outcomes regarding the osteo- and angiogenic effects of NnHap on rBMSCs and hUVECs while also indirectly facilitating bone repair through macrophage-mediated immunomodulatory microenvironments. An in vivo study was conducted to further substantiate the beneficial effects of NnHap.

#### Micro-CT analysis

**Fig. 9.**
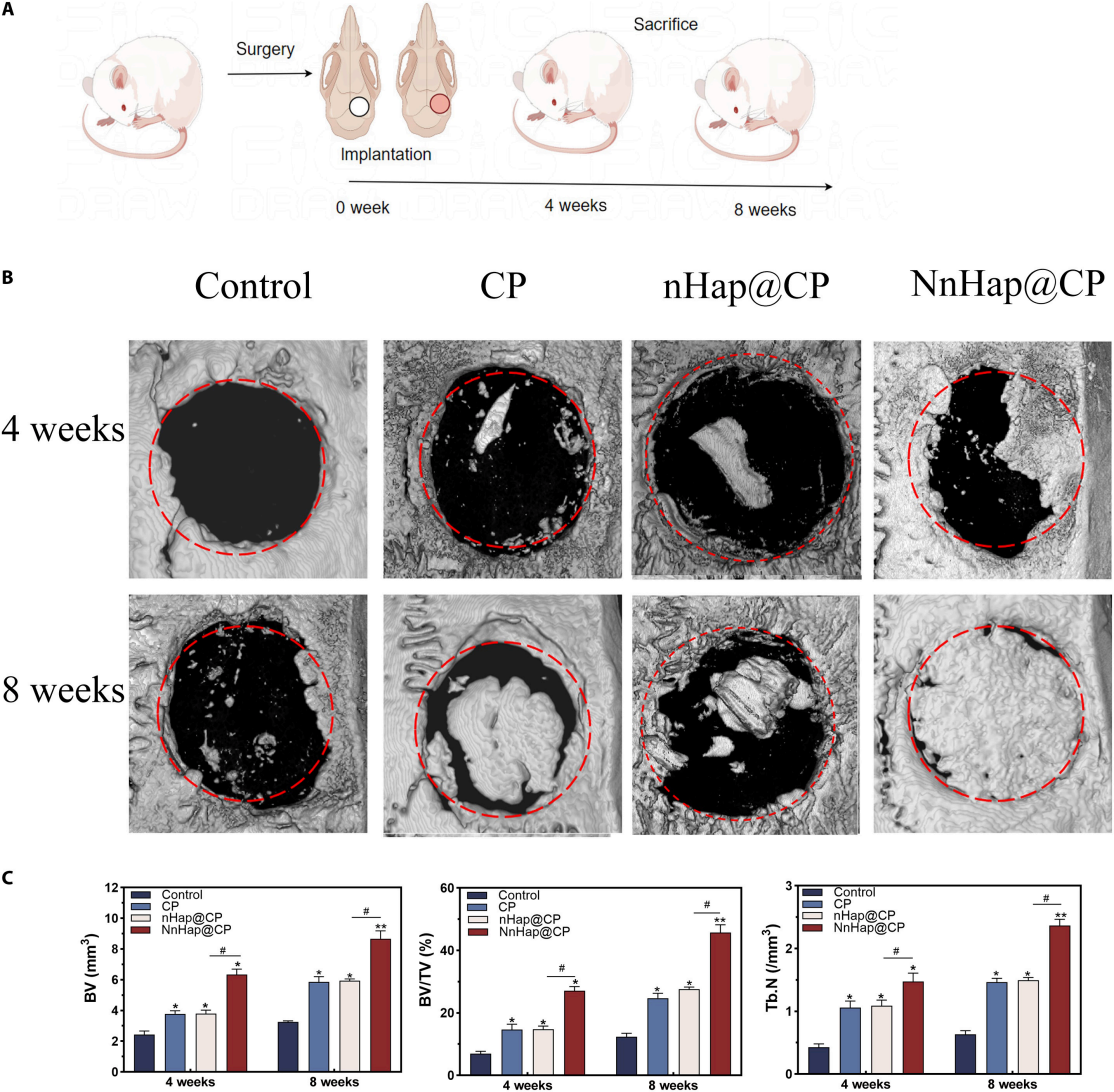
The results of in vivo study. (A) The schematic diagram of the animal experiment procedure; (B) the micro-computed tomography (micro-CT) 3-dimensional (3D)-reconstructed models; (C) the results of bone volume, bone volume/total volume ratio, and trabecular bone number of the 4 groups. Results are represented as the mean ± SD of 3 independent experiments (*n* = 4); for statistical analysis, a 2-way ANOVA test was used to compare groups across 4 and 8 weeks. Significant differences: *P < 0.05; ^#^*P* < 0.05; **P < 0.01. BV, bone volume; BV/TV, bone volume/tissue volume ratio; TB.N, trabecular bone number.

The bone repair capability of NnHap@CP scaffolds was evaluated by implanting them in the rat calvarial defect model, as shown in Fig. 9A. The micro-CT 3D-reconstructed models of the calvarial defects demonstrated that the NnHap@CP group exhibited the most robust capacity for new bone formation among the 4 groups (Fig. 9B). Furthermore, the results regarding BV, BV/TV ratio, and Tb.N were consistent with the 3D reconstructions presented in Fig. 9C. Precisely, at 4 weeks, the NnHap@CP group showed significantly better outcomes than the nHap@CP, CP, and control groups (BV: 6.3 ± 0.36 mm^3^ vs 3.9 ± 0.31 mm^3^ vs 3.8 ± 0.2 mm^3^ vs 2.4 ± 0.23 mm^3^, *P* < 0.05; BV/TV: 27.1 ± 1.34 vs 14.8 ± 1.9 vs 14.7 ± 1.74 vs 6.9 ± 0.78, *P* < 0.05; Tb.N: 1.5 ± 0.13 mm^3^ vs 1.1 ± 0.11 mm^3^ vs 0.4 ± 0.06 mm^3^, *P* < 0.05). Similar outcomes were also observed at 8 weeks (BV: 8.7 ± 0.51 mm^3^ vs 6.1 ± 0.39 mm^3^ vs 5.9 ± 0.35 mm^3^ vs 3.3 ± 0.08 mm^3^, *P* < 0.05; BV/TV: 45.7 ± 2.49 vs 26.2 ± 1.81 vs 24.7 ± 1.53 vs 12.4 ± 1.08, *P* < 0.05; Tb.N: 2.4 ± 0.09 mm^3^ vs 1.5 ± 0.06 mm^3^ vs 1.5 ± 0.06 mm^3^ vs 0.6 ± 0.06 mm^3^, *P* < 0.05), and a 2-way ANOVA test was applied to compare groups across 4 and 8 weeks. Those results indicate new bone formation across the 4 groups.

#### H&E staining and Masson’s trichrome staining

**Fig. 10.**
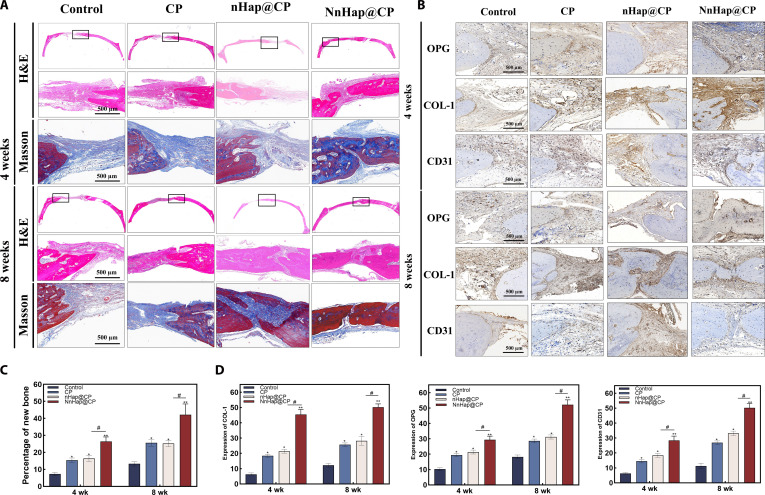
The results of specimen tissue sections under hematoxylin and eosin (H&E), Masson’s trichrome, and immunohistochemical staining. (A) The H&E and Masson’s staining of specimen sections (scale bars: 500 μm); (B) the immunohistochemical staining of specimen sections (scale bars: 500 μm); (C) the percentage of new bone; (D) the results of OPG, COL-1, and CD31 of the 4 groups. Results are represented as the mean ± SD of 4 independent experiments (*n* = 4); for statistical analysis, a 2-way ANOVA test was applied to all panels that compare groups across 4 and 8 weeks. Significant differences: *P < 0.05; ^#^*P* < 0.05; **P < 0.01.

The results demonstrated that only a few areas of new bone formation and neovascularization were observed at the edge of the defect in the control group, due to overlying fibrous tissue collapsing into the bone defect region. The NnHap@CP group achieved significantly better bone regeneration and neovascularization than the CP and control groups, suggesting that the NnHap@CP scaffolds can promote bone repair and blood vessel formation (Fig. 10A), and the quantitative results of new bone are listed in Fig. 10C.

#### Immunohistochemical staining

Immunohistochemical staining was also conducted after the tissue specimens had been decalcified and sectioned at 4 and 8 weeks postimplantation. Osteogenic markers (OPG and COL-1) and an angiogenic marker (CD31) were used to evaluate the osteo- and angiogenesis effects of the NnHap@CP scaffold in vivo (Fig. 10B). The highest expressions of COL-1, OPG, and CD31 were observed in the NnHap@CP group (Fig. 10D). In contrast, the weakest was observed in the control group. It is thus demonstrated that NnHap@CP effectively improves osteogenesis and neovascularization, as well as activates macrophage polarization and manages bone immunomodulation.

#### Matrigel plug assay

The angiogenesis effect of the NnHap@CP scaffold was assessed in vivo. It can be seen from Fig. S12A that the hemoglobin content was highest in the NnHap@CP group, which was significantly higher than those of the other groups (Fig. S12C). Similarly, the immunohistochemical staining (CD31) of those sections was also analyzed (Fig. S12B), and the highest expression level of CD31 was observed in the NnHap@CP group (Fig. S12D).

#### Macrophage polarization in vivo

To evaluate whether stable M2 polarization persists in vivo, immunofluorescence of skull specimens was performed (Fig. S13A). As shown in Fig. S13B, there were obviously more CD206-positive cells in the NnHap@CP group than in the other groups. Those results reveal that stable and effective M2 macrophage polarization existed in vivo.

## Conclusion

Our study focused on designing and fabricating an efficient composite scaffold containing NnHap, aiming to analyze its effects on osteo-/angiogenesis and bone immunomodulation. The findings demonstrated that NnHap@CP scaffolds can enhance angiogenesis in hUVECs, reprogram macrophages to the M2 phenotype, and stimulate osteogenesis in rBMSCs by up-regulating the OPG/RANK/RANKL signaling pathway. Furthermore, we demonstrated that the NnHap@CP scaffold can promote in situ new bone formation by osteoinduction and bone immunomodulation. Above all, NnHap can improve bone regeneration, both directly through biochemical action and indirectly by bone immunomodulation, via macrophage polarization, which could be a promising strategy for treating critical bone defects. However, additional related studies are required to validate the effects of the NnHap@CP scaffolds.

### Limitation

(a) Evaluating our scaffold in a long-bone defect model and in larger animals is a critical next step to strengthen the translational relevance of our work. (b) The tunability of this scaffold is also important, which makes it possible to be used for different defect sites (weight-bearing bone, like femur and tibia). The tunability of our hydrogel may be achievable. (1) For non-load-bearing sites (maxillofacial defects), we can increase the degradation rate by reducing the cross-linking density from PLAD to CMCS using a lower-molecular-weight CMCS. (2) For larger, load-bearing long-bone defects (femoral defects), which require longer mechanical support, we can slow down the degradation by increasing the cross-linking density and incorporating more hydrophobic components. (c) The lack of longer-term osteogenesis data (such as ARS and ALP staining at day 32 or later) is a limitation of our study.

## Ethical Approval

The present study was approved by the Institutional Review Boards and Ethics Committee of Ningbo University (No. NBU20240318).

## Data Availability

The data that support this study are available from the corresponding authors upon reasonable request.
