# Supplementary material for: A Needlelike Nano-hydroxyapatite-Based Hydrogel Accelerates Critical Bone Defect Regeneration via Osteo-/Angiogenesis and Osteoimmune Regulation
Source: Biomater Res. 2026 Apr 2;30:0344. doi: 10.34133/bmr.0344 (PMC13044407; doi:10.34133/bmr.0344)
Supplement: Supplementary 1 — Figs. S1 to S13 Table S1 [file bmr.0344.f1.doc]

Needle-like nano hydroxyapatite based composite hydrogel accelerate critical bone defect regeneration via osteo-/angiogenesis and osteoimmune regulation

**Materials and regents**

calcium nitrate tetrahydrate was provided by Sigma Inc. (Unna, Germany). diammonium hydrogen phosphate was provided by Aladdin Inc (Shanghai, China). Urea was provided by Sigma Inc. (Unna, Germany). mercaptopropionic acid was provided by Sigma-Aladdin Inc. (Missouri, USA), methylene dichloride provided by Sigma Inc. (Unna, Germany), carboxymethyl chitosan provided by Sigma-Aladdin Inc. (Missouri, USA), polylactic acid derivatives was provided by Daigang Biology(Shandong, China).Dulbecco’s modified Eagle’s medium (DMEM), α-mem,0.25% trypsin-EDTA, phosphate buffer saline (PBS), and penicillin/streptomycin (P/S) were produced by Gibco (CA, USA). Tetracycline (TE), alizarin red (AL) were supplied by Sigma-Aldrich (Missouri, USA). Rat bone marrow-derived mesenchymal stem cells and RAW 264.7 cell were supplied by Pricella, (Wuhan, China), Human umbilical vein endothelial cells were supplied by Meisen (Zhejiang, China) ECM was supplied by ScienCell, (Los Angeles, USA). calcein acetoxymethyl ester (AM) /propidium iodide (PI) was supplied by Elabscience, (Wuhan, China). CCK-8 was supplied by Abcam (Cambridge, England). alizarin red staining solution was supplied by Solarbio (Beijing, China). cetylpyridinium chloride was provided by Sigma-Aladdin Inc. (Missouri, USA), ALP staining working solution and ALP activity assay working solution were peovided by Beyotime (Chengdu, China), 2-(4-Amidinophenyl)-6-indolecarbamidine dihydrochloride (DAPI), Actin-tracker green-488, radio-immunoprecipitation (RIPA) lysis buffer and Bovine Serum Albumin (BSA) were manufactured by Beyotime (Shanghai, China).


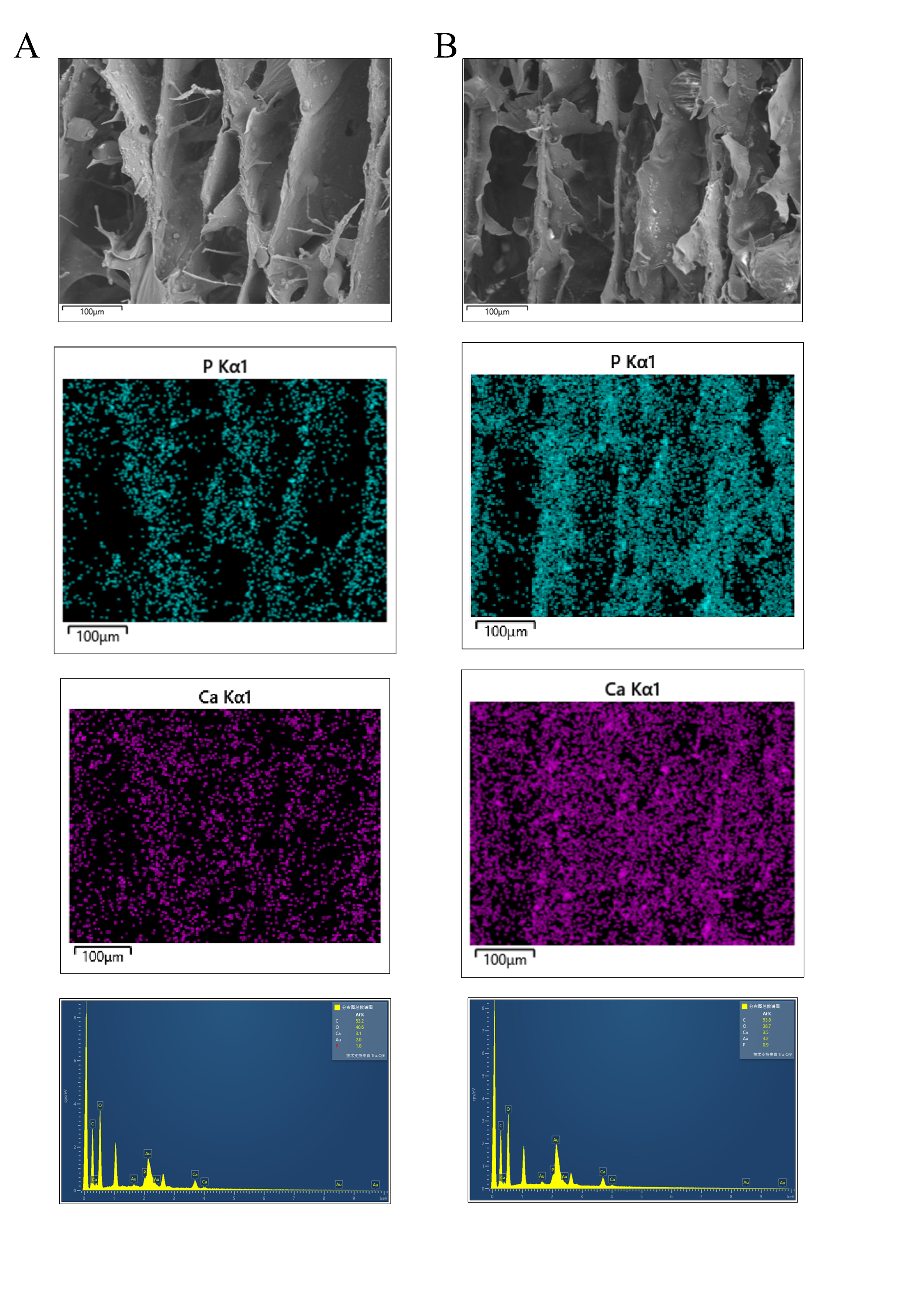


Fig S1 A. The eds Mapping result of NnHap@CPscaffold; B. The eds Mapping result of nHap@CP scaffold


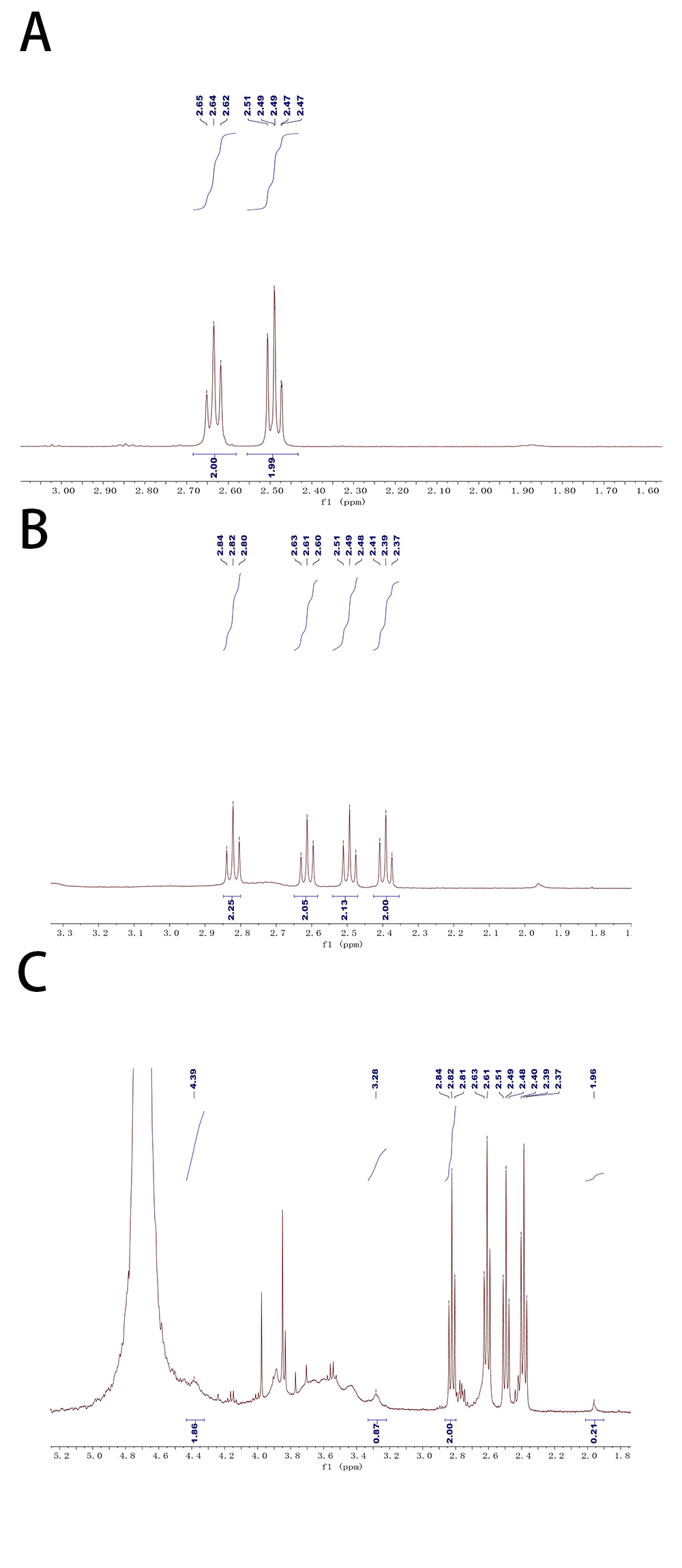


Figure S2. measure the graft copolymerization rate by 1H NMR spectral. A.1H NMR spectral of mercaptopropionic acid.The t peak at 2.64 ppm is the methylene hydrogen adjacent to the mercapto group. B.1H NMR spectral of MA-modified NnHap. The t peak at 2.82 ppm and 2.39 ppm represent the content of the MA that have been grafted. C.1H NMR spectral of NnHap@cp scaffolds. The t peak at 2.82 ppm represent the content of the MA-modified NnHap that have been grafted. And the grafting rate was found to be 43.8%.


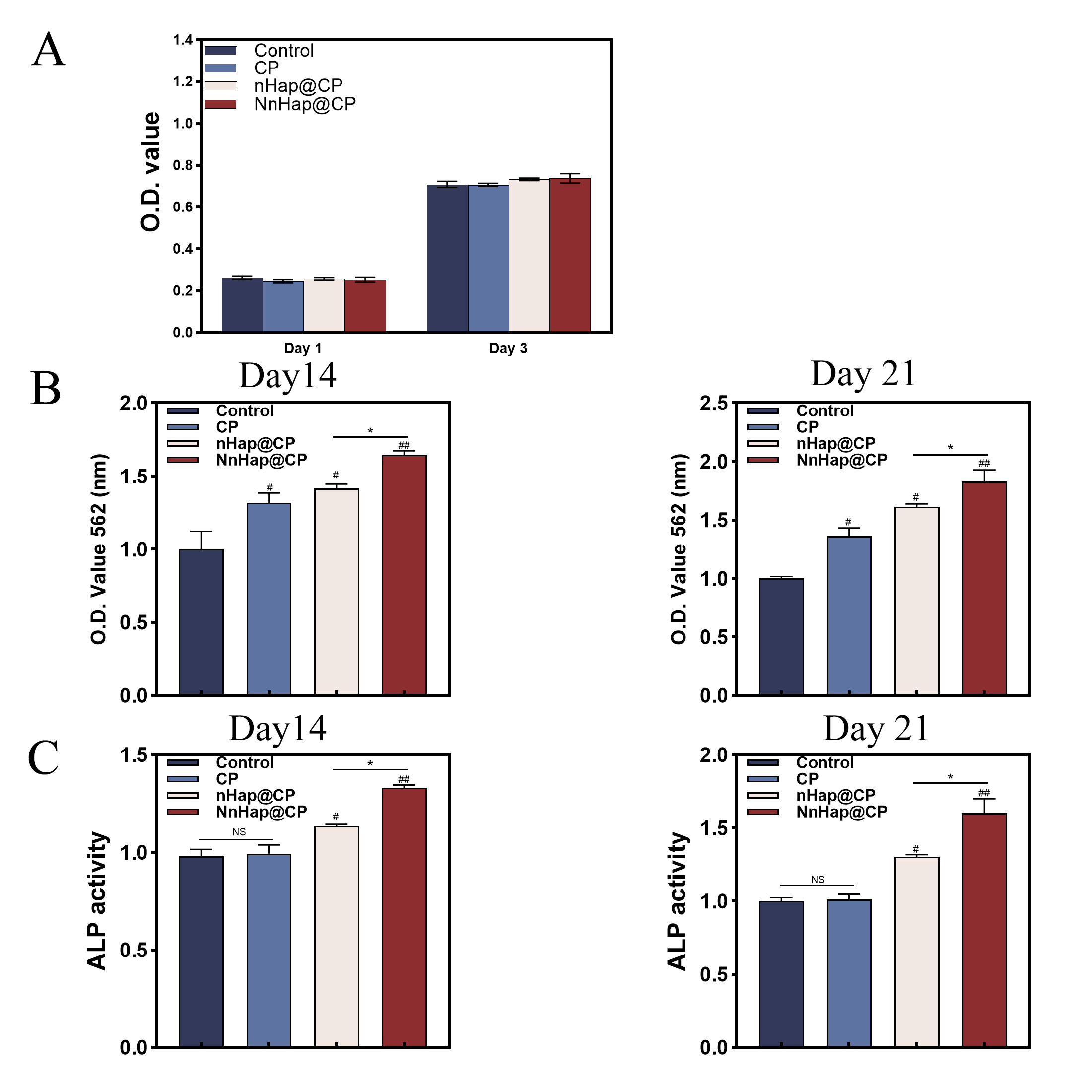


Figure S3 A.The CCK-8 result of BMSCs after co-culture after 3 and 5 day;B.The O.D. value result of ARS after dissolved with 10% cetylpyridinium chloride at 14 and 21 day;C. The alp activities result of BMSCs after 14 and 21 day.


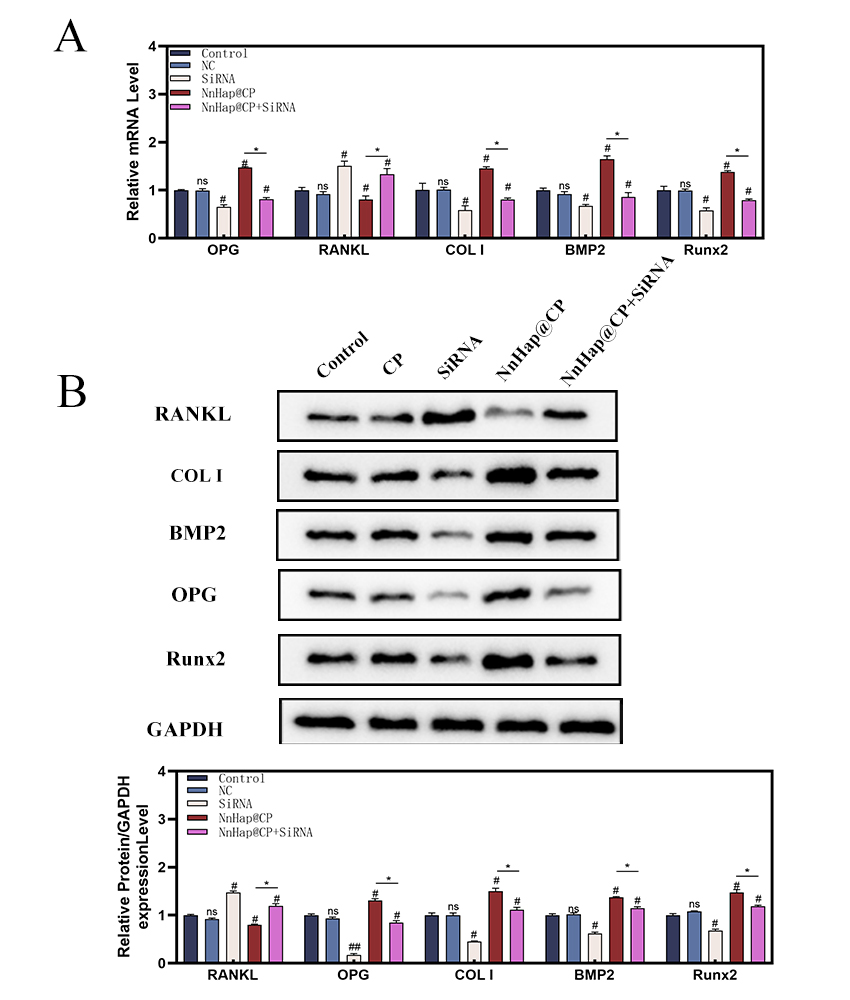


Figure S4 The results of PCR and WB after inhibit OPG by SiRNA. A.qRT-PCR of BMP2, COL-I, Runx2, OPG and RANKL of Control, NC, SiRNA, NnHap@CP and NnHap@CP+SiRNA group after 1 week. B. WB of BMP2, COL-I, Runx2, OPG and RANKL of Control, NC, SiRNA, NnHap@CP and NnHap@CP+SiRNA group after 1 week. C. quantitative results of WB. For statistical analysis, a one‐way ANOVA test was used. Significant differences: *p < 0.05,#p<0.05,##p < 0.01.


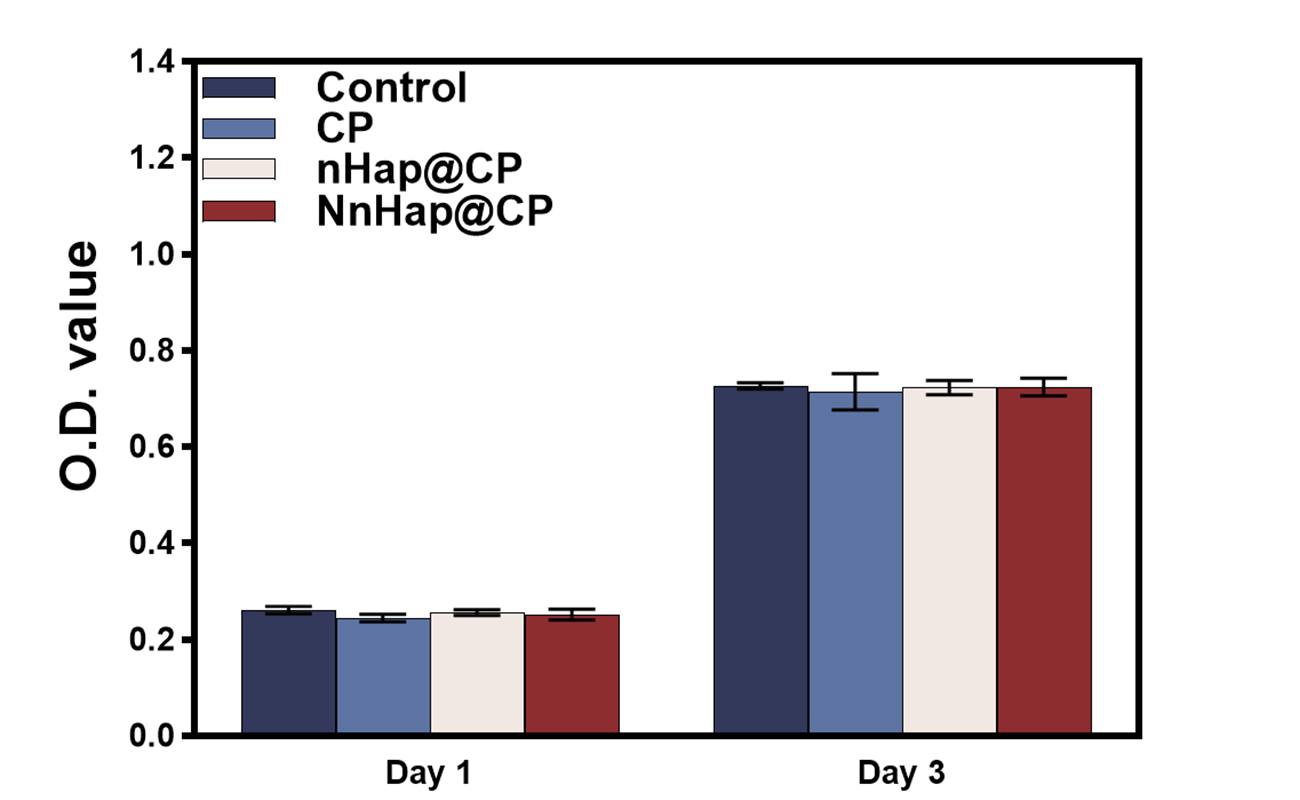
Figure S5 A.The CCK-8 result of macrophage after co-culture after 3 and 5 day.


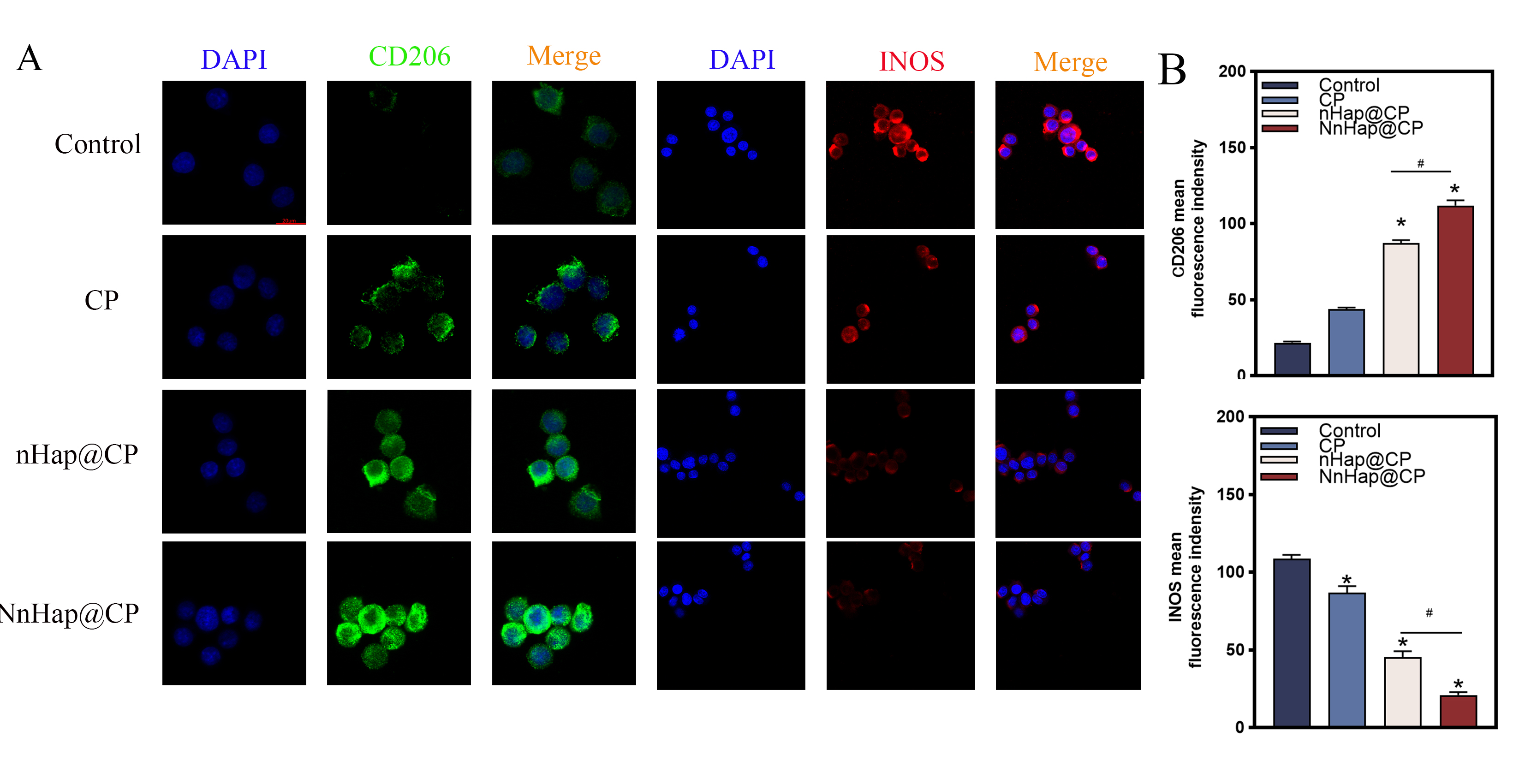


Figure S6 The effect of NnHap@CP scaffolds on macrophage. A.The immunofluorescence staining of macrophage(lower; scale bars: 100 µm); B The fuorescence intensity of immunofluorescence staining. Results are represented as the mean ± SD of three independent experiments (n = 3), for statistical analysis, a one‐way ANOVA test was used. Significant differences: *p < 0.05,#p<0.05,##p < 0.01.
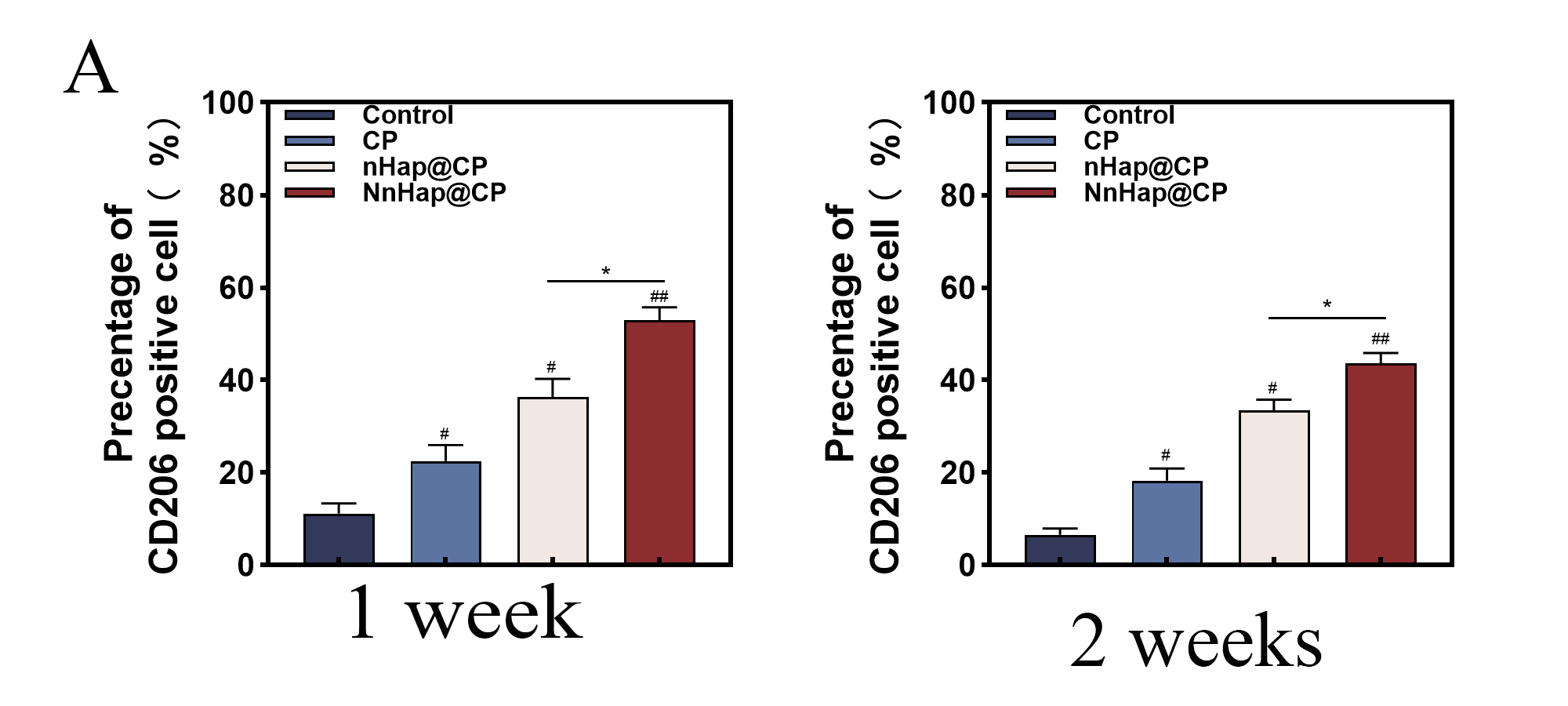


Figure S7. The quantitative results of flow cytometry. Highest CD 206 expression (M2 polarization) in NnHap@CP group were observed at 1 and 2 weeks.


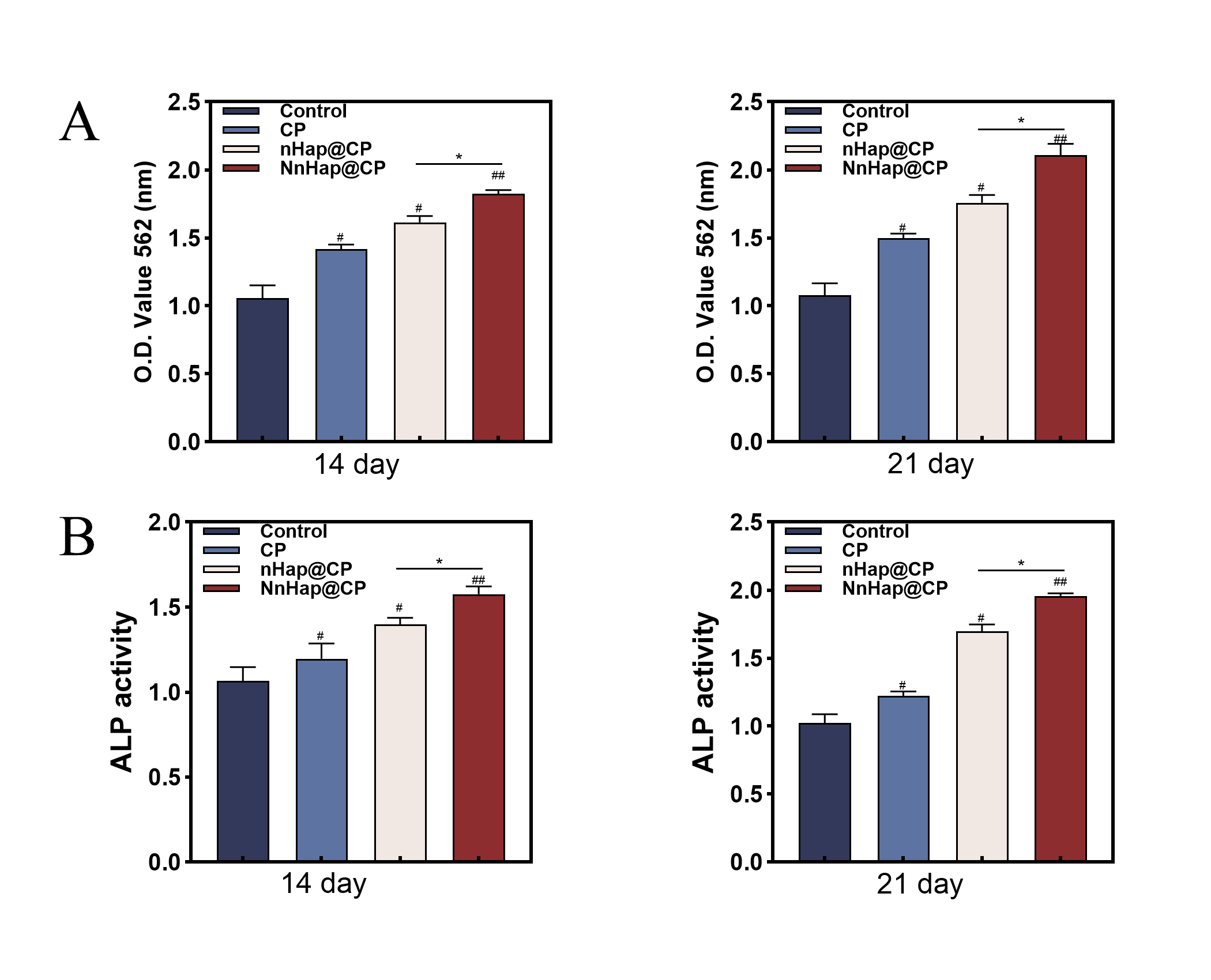


Figure S8 A. The O.D. value result of ARS after dissolved with 10% cetylpyridinium chloride at 14 and 21 days.B. The alp activities res after 14 and 21 days.


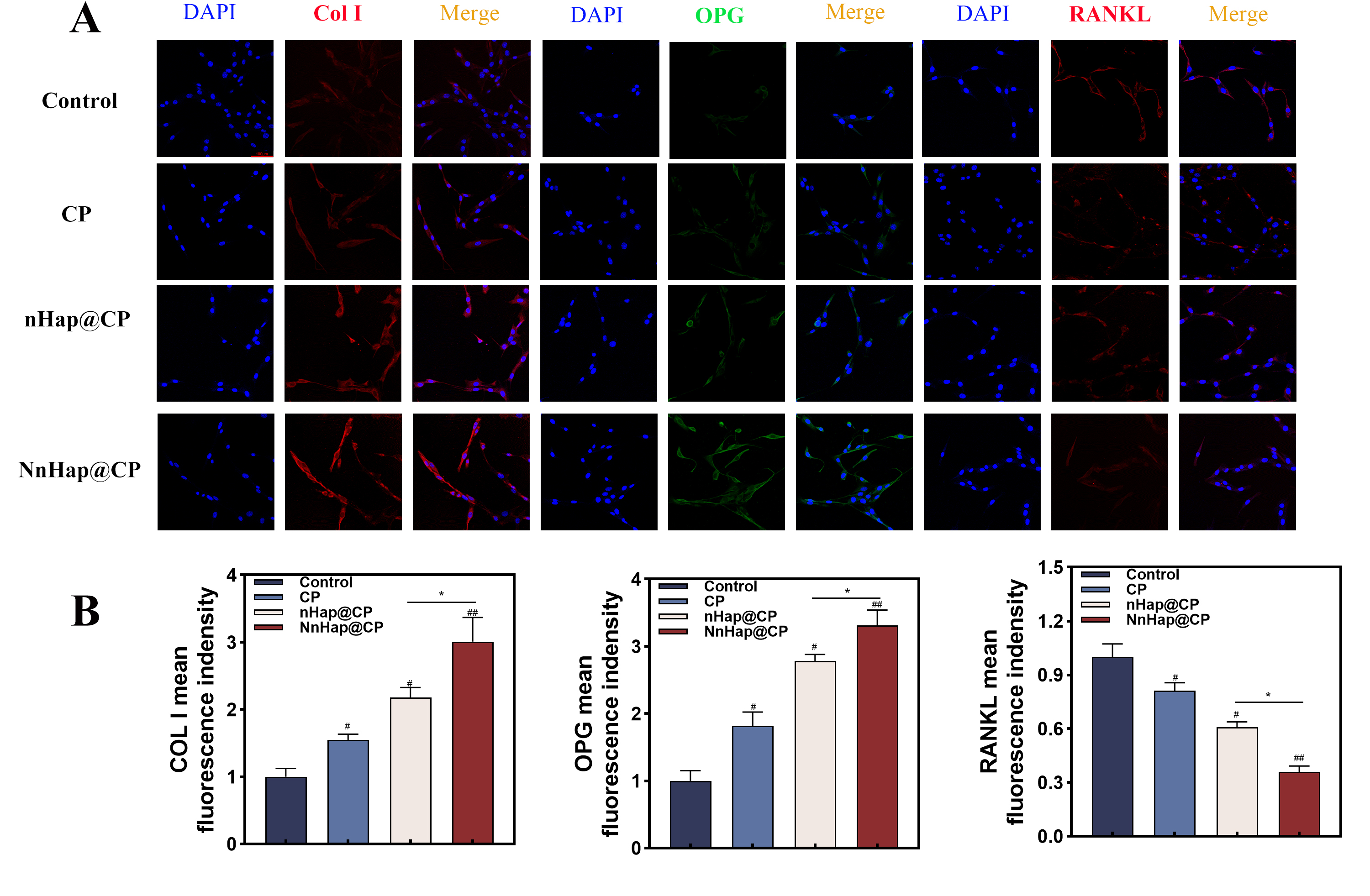


Figure S9 The effect of co-culture media of macrophage on rBMSCs osteogenesis. A The immunofluorescence staining of rBMSC with CM after 1 week; B The fuorescence intensity of each immunofluorescence staining(lower; scale bars: 100 µm), results are represented as the mean ± SD of three independent experiments (n = 3), for statistical analysis, a one‐way ANOVA test was used. Significant differences: *p < 0.05,#p<0.05,##p < 0.01.


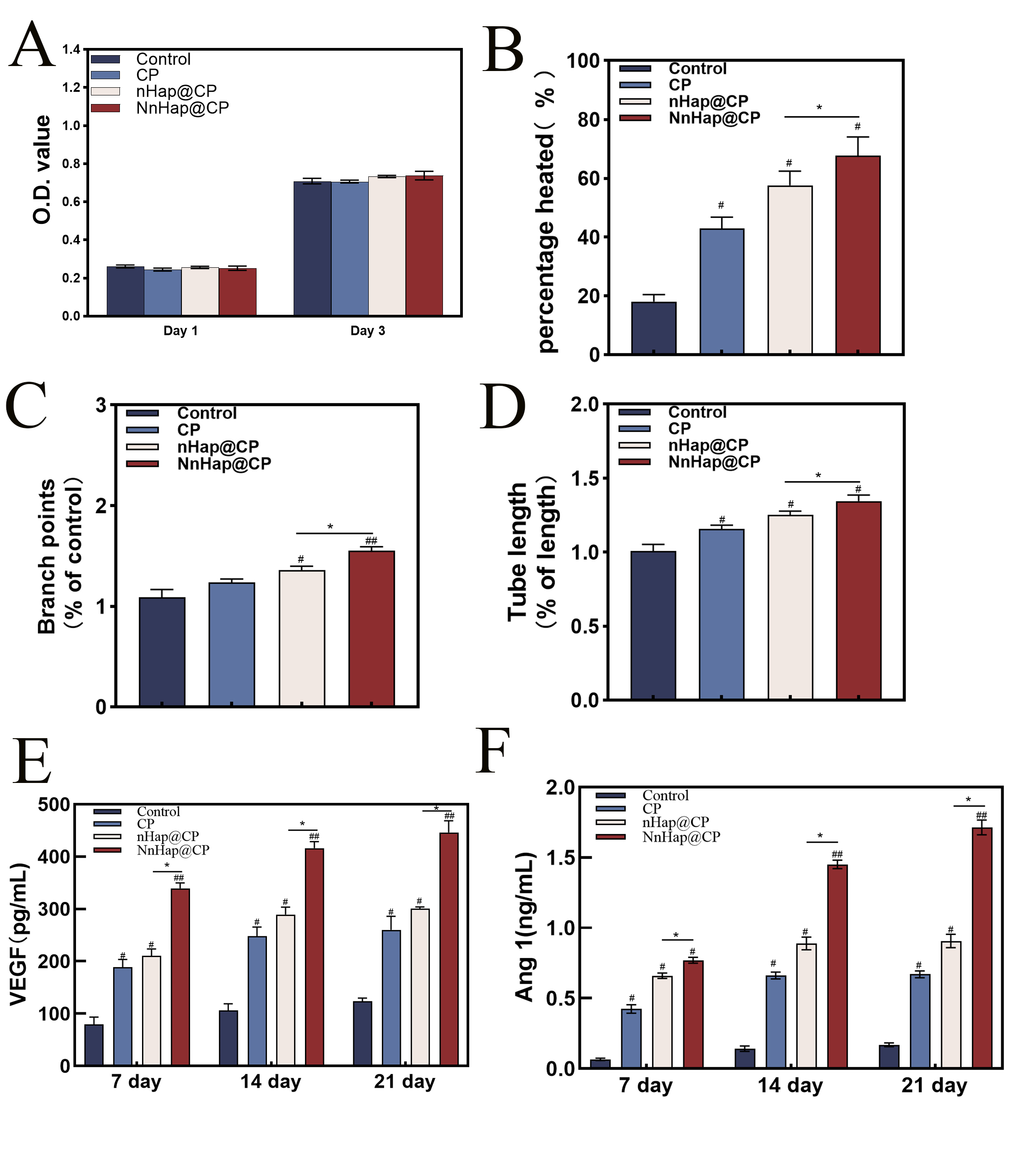
Figure S10 A.The CCK-8 result of hUVECS after co-culture after 1 and 3 day B the quantitative analysis of scratchassay. C . the tube length and branch point of three groups in Tube formation assay；C ELISA of VEGF and Ang1 of the macrophage-conditioned media (CM) at 7, 14 and 21 days.


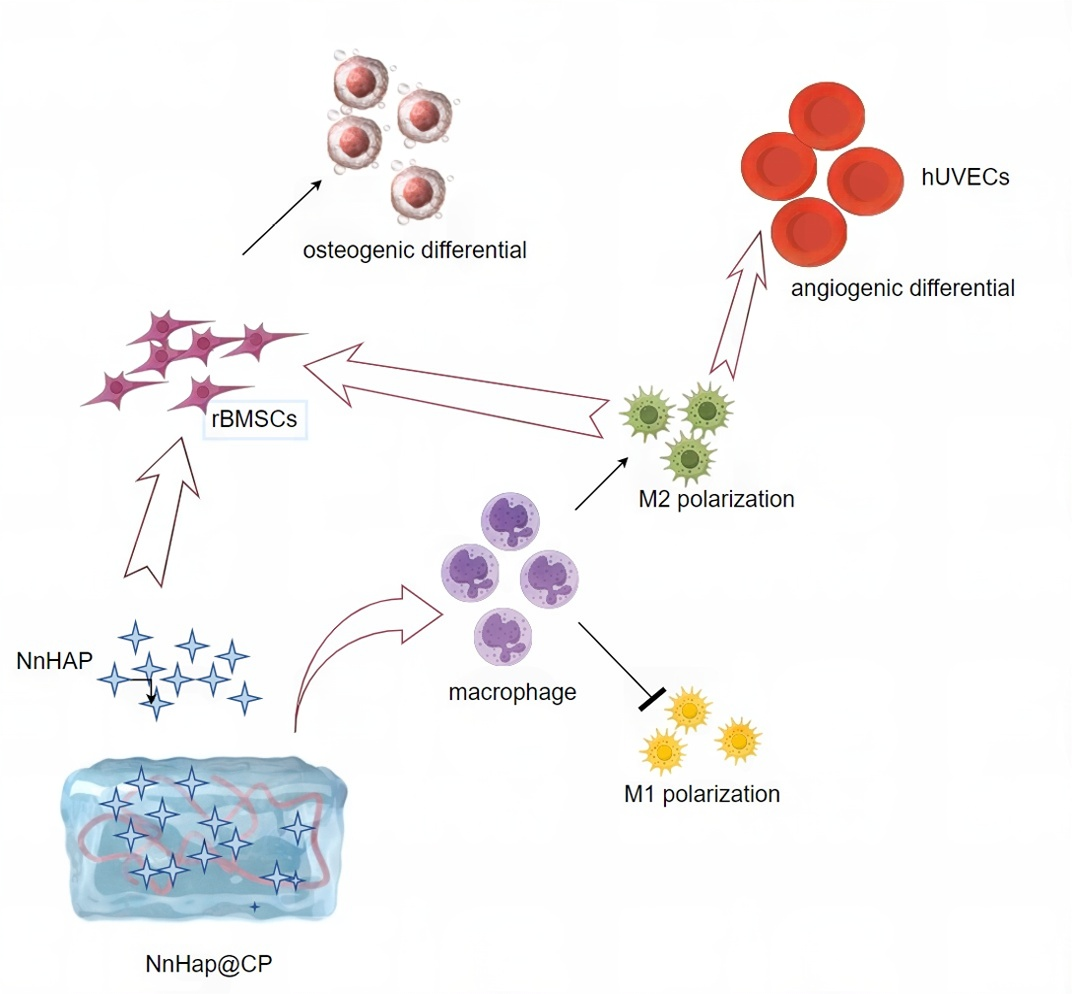


Figure S11 The graphic abstract of *vitro* study.


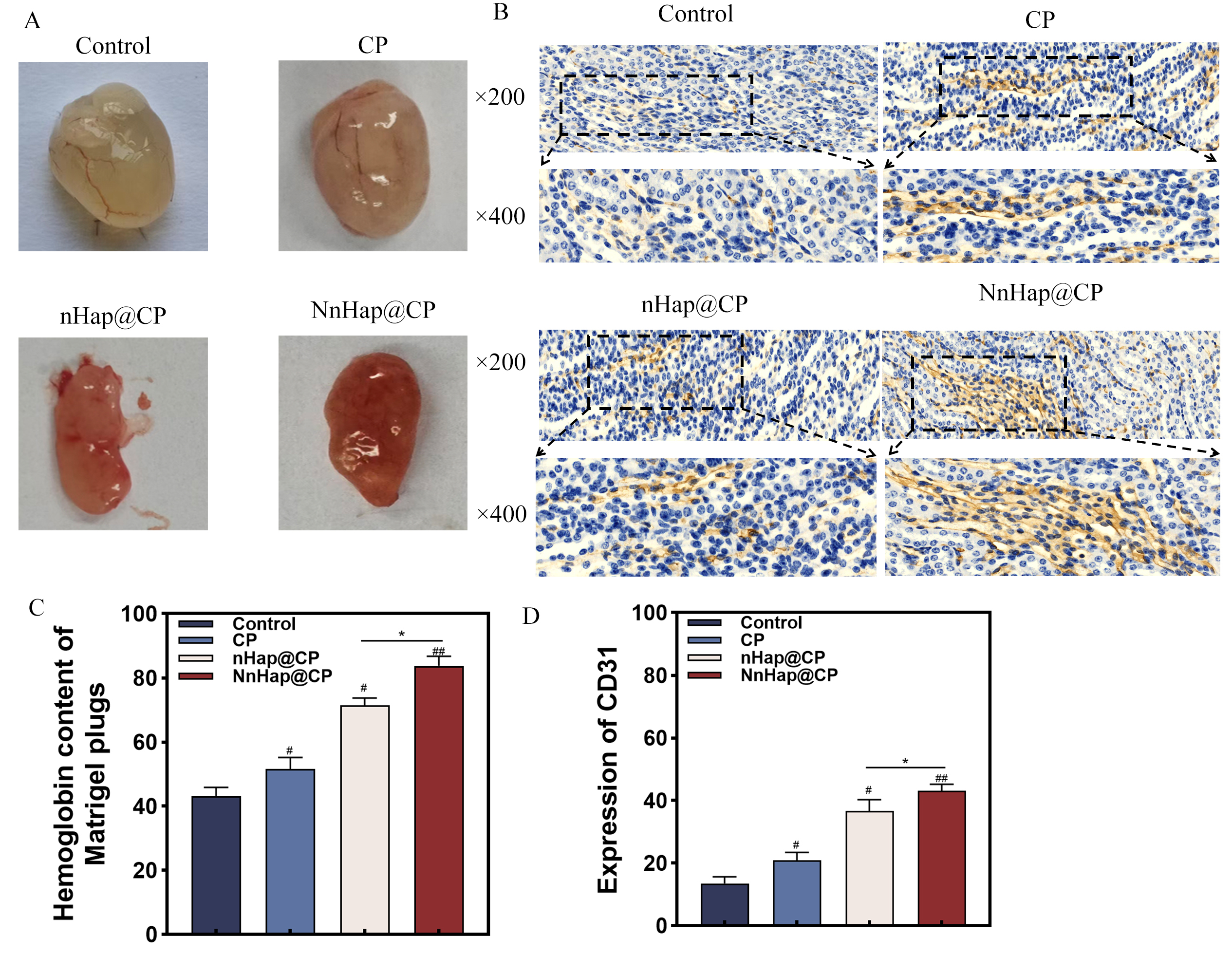


Figure S12 The results of angiogenesis in*Vivo*. A. gross images of Matrigel plugs of four groups.B. The CD31 Immunohistochemical staining images of sections of Matrigel plugs at day 7 of the in vivo Matrigel plug assay. C The Hemoglobin content in Matrigel plugs. D. quantitative results of CD31 Immunohistochemical staining.


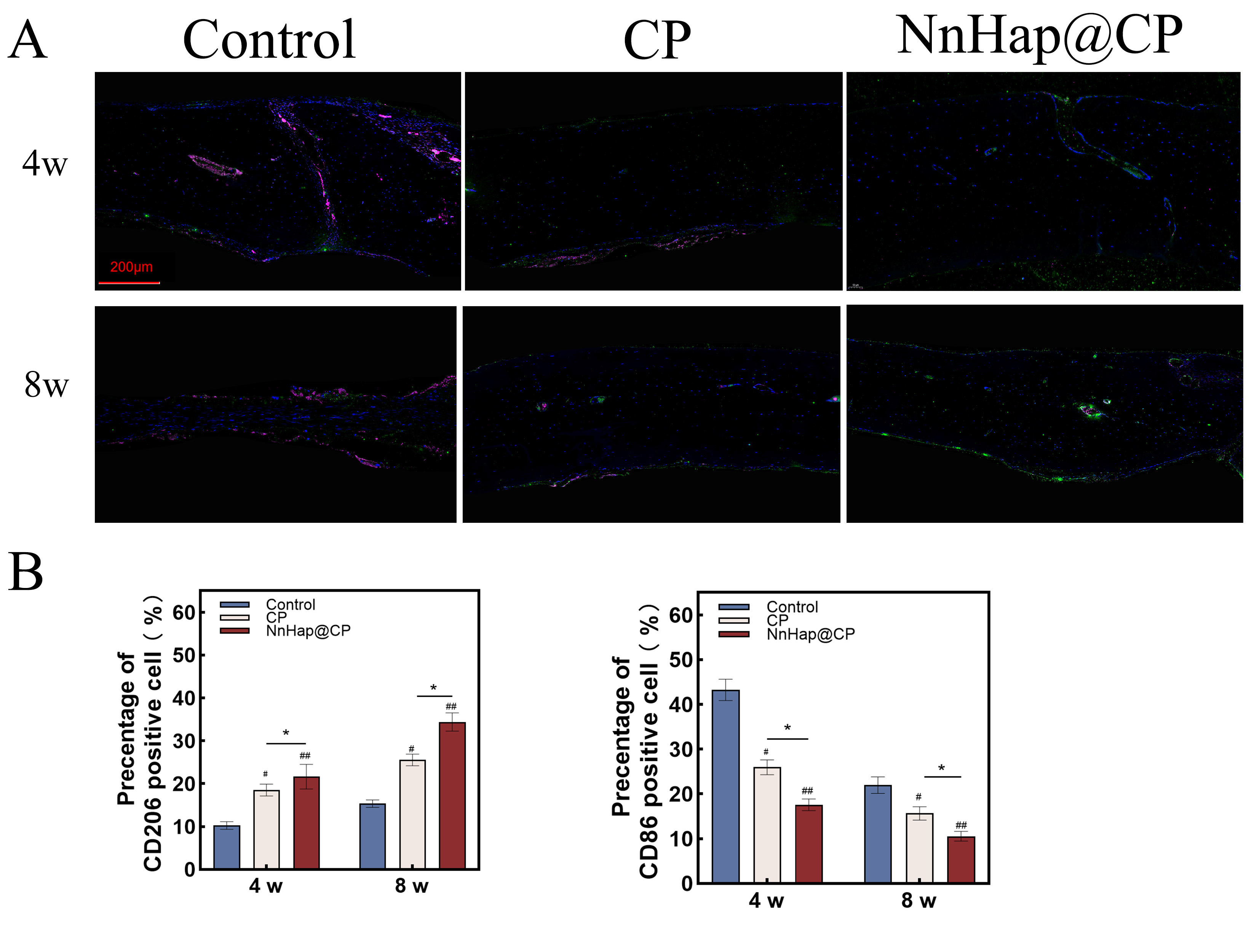


Figure S13 The results of macrophage polarization in*Vivo*. A.The Immunofluorescence of Skull specimen (CD206 Green 488nm; CD86 Red 596nm). E the quantitative analyze of the percentage of CD206-Positive and CD86-Positive cells in these groups.

**Table S1** The sequences of genes (F: forward primer R: reverse primer).

| *Gene* | Primer Sequences (5'-3') |
| --- | --- |
| *COL-1* | F:5'- CATGAGCCGAAGCTAACCCC -3' |
|  | R:5'- GCTGACTTCAGGGATGTCTTCT -3' |
| *RUNX-2* | F:5'- CACAAGTGCGGTGCAAACTT -3' |
|  | R:5'- AATGACTCGGTTGGTCTCGG -3' |
| *BMP-2* | F:5'- TGCGGTCTCCTAAAGGTCG -3' |
|  | R:5'- CACTAGAAGACAGCGGGTCC -3' |
| *OPN* | F:5'- CCAGCCAAGGACCAACTACA -3' |
|  | R:5'- AGTGTTTGCTGTAATGCGCC -3' |
| *ALP* | F:5'- GCTGGCAAAATACCAGGGGA -3' |
|  | R:5'- TGAGGCGTTTCGGTTGACAT -3' |
| *VEGF* | F:5'- CAACAAATGTGAATGCAGACCAA -3' |
|  | R:5'- GCTCCAGGGCATTAGACAGC -3' |
| *CD31* | F:5'- GGAAAGCTGTCCCTGATGCC -3' |
|  | R:5'- CAAGGGAGCCTTCCGTTCTA -3' |
| *IL-1β* | F:5'- CAGCTTTCGACAGTGAGGAGA -3' |
|  | R:5'- TTGTCGAGATGCTGCTGTGA -3' |
| *IL-6* | F:5'- TCCATCTGCCCTTCAGGAAC -3' |
|  | R:5'- GGCTGGAAGTCTCTTGCGG -3' |
| *IL-10* | F:5'- CCTCTGGATACAGCTGCGAC -3' |
|  | R:5'- AGACACCTTTGTCTTGGAGCTTA -3' |
| *TGF-β* | F:5'- GACCGCAACAACGCAATCTA -3' |
|  | R:5'- CGTGTTGCTCCACAGTTGAC -3' |
| *OPG* | F:5'- CTCACTTGGCCTCCTGCTAA -3' |
|  | R:5'- TCGCACAGGGTGACATCTAT -3' |
| *RANKL* | F:5'- GCCGTGCAAAGGGAATTACA -3' |
|  | R:5'- GAGCCACGAACCTTCCATCA -3' |
| *GAPDH* | F:5'- AGTGCCAGCCTCGTCTCATA -3' |
